# Hurricane *Isaac* brings more than oil ashore: Characteristics of beach deposits following the *Deepwater Horizon* spill

**DOI:** 10.1371/journal.pone.0213464

**Published:** 2019-03-18

**Authors:** Karin L. Lemkau, Christopher M. Reddy, Catherine A. Carmichael, Christoph Aeppli, Robert F. Swarthout, Helen K. White

**Affiliations:** 1 Department of Marine Chemistry and Geochemistry, Woods Hole Oceanographic Institution, Woods Hole, Massachusetts, United States of America; 2 Bigelow Laboratory for Ocean Sciences, East Boothbay, Maine, United States of America; 3 Department of Chemistry and Environmental Science Program, Appalachian State University, Boone, North Carolina, United States of America; 4 Department of Chemistry, Haverford College, Haverford, Pennsylvania, United States of America; University of Houston, UNITED STATES

## Abstract

Prior to Hurricane *Isaac* making landfall along the Gulf of Mexico coast in August 2012, local and state officials were concerned that the hurricane would mobilize submerged oiled-materials from the *Deepwater Horizon* (DWH) spill. In this study, we investigated materials washed ashore following the hurricane to determine if it affected the chemical composition or density of oil-containing sand patties regularly found on Gulf Coast beaches. While small changes in sand patty density were observed in samples collected before and after the hurricane, these variations appear to have been driven by differences in sampling location and not linked to the passing of Hurricane *Isaac*. Visual and chemical analysis of sand patties confirmed that the contents was consistent with oil from the Macondo well. Petroleum hydrocarbon signatures of samples collected before and after the hurricane showed no notable changes. In the days following Hurricane *Isaac*, dark-colored mats were also found on the beach in Fort Morgan, AL, and community reports speculated that these mats contained oil from the DWH spill. Chemical analysis of these mat samples identified *n*-alkanes but no other petroleum hydrocarbons. Bulk and δ^13^C organic carbon analyses indicated mat samples were comprised of marshland peat and not related to the DWH spill. This research indicates that Hurricane *Isaac* did not result in a notable change the composition of oil delivered to beaches at the investigated field sites. This study underscores the need for improved communications with interested stakeholders regarding how to differentiate oiled from non-oiled materials. This is especially important given the high cost of removing oiled debris and the increasing likelihood of false positives as oiled-materials washing ashore from a spill become less abundant over time.

## Introduction

The 2010 *Deepwater Horizon* (DWH) disaster resulted in the release of 4.1 million barrels of oil from the Macondo well (MW) [1], contaminating over 1700 km of the Gulf of Mexico coast [2,3] including beaches [4,5] and salt marshes [6–10]. The spill had substantial economic impacts on Gulf Coast communities and fisheries [11–13]. While extensive response activities were conducted in the months and years following the spill, not all residual oil from the event has been removed from the environment [14]. Residual oiled material exists in the surf zone in the form of submerged oil mats and is regularly deposited on beaches [4,15–18,2]. In the years since the spill, discrete areas of shoreline have experienced periodic remobilization of weathered oil and sand mixtures or “sand patties”, also known as surface residual balls [4,19,20]. These are believed to originate from submerged oil mats located offshore [2]. Monitoring the composition of these sand patties has shown changes in their composition since the spill, including formation of environmentally recalcitrant oxygenated hydrocarbons [19,21]. Ongoing field investigations of oil from the DWH spill in the coastal environment can provide useful information to inform future cleanup efforts.

Storm events are a particular concern for oil remobilization due to their high energy and dynamic nature. Storm surges, high winds, and heavy rainfall associated with hurricanes can restructure coastlines through the movement of sands and sediments, remobilizing submerged oil residues [22]. For example, following Tropical Storm *Lee* in 2011, “tar mat fragments” containing oil residues from the DWH spill were deposited onto Alabama beaches, presumably from submerged oil mats offshore [17,23].

Prior to Hurricane *Isaac’s* landfall (August 29, 2012), elected officials and the general public expected the hurricane would expose buried DWH-derived oiled materials or remobilize oiled materials within the sub-tidal zone, potentially harming both coastal ecosystems and coastal economies. [24–26]. As a response, 565,000 pounds (256,000 kg) of suspected oiled materials were recovered from Gulf Coast beaches in the month following the hurricane [27]. However, the extent to which recovered beach materials were oiled and/or directly related to the DWH spill is unknown. Frequently oiled debris from shorelines is too contaminated with beach material (e.g. sand, rocks, biomass, plastic etc.), or funding and/or resources limited, such that stabilization, or recycling and reprocessing to recover the oil is impractical. Instead this waste is often shipped to landfills or disposed of through incineration [28]. The DWH spill produced over 89,000 tonnes (89,000,000 kg) of solid waste [29]. These disposal costs can be a major portion of total cleanup costs and the first step in controlling this cost is accurate identification of oiled materials [28].

To assess the impact of Hurricane *Isaac* on oiled materials washed ashore, we studied physical and chemical properties of samples collected from several Gulf Coast beaches before and after the hurricane. We were uniquely positioned to undertake this study due to our ongoing sampling efforts subsequent to the DWH spill. These sampling efforts have involved collection of hundreds of samples many of which have been linked to the DWH spill through biomarker analysis [19,21,30,31]. Also, we had collected samples at storm-affected sites in the weeks and days before the hurricane. Two days after the storm made landfall we returned to the previously sampled locations and collected post-storm samples. Specifically, the research reported here focuses on samples collected before and after the hurricane at Fort Morgan, AL.

Sand patties commonly found on Gulf Coast beaches after the spill and those collected just prior to Hurricane *Isaac* were visually consistent with those collected immediately following the hurricane. We hypothesized that a high energy storm event, such as a hurricane, would potentially mobilize sand patties of higher density and from deeper sources than those typically washing ashore. While oiled materials would likely still be related to the DWH spill, movement of submerged oil mats may result in changes to the composition of sand patties being washed ashore. This had previously been observed in samples collected following tropical storm *Lee*, which contained abundant petroleum-derived *n*-alkanes, indicative of less weathered oil [17,23]. To investigate if the hurricane delivered materials of higher density or with different degrees of weathering we compared density and chemical composition of sand patties collected before and after the hurricane.

**Fig 1:**
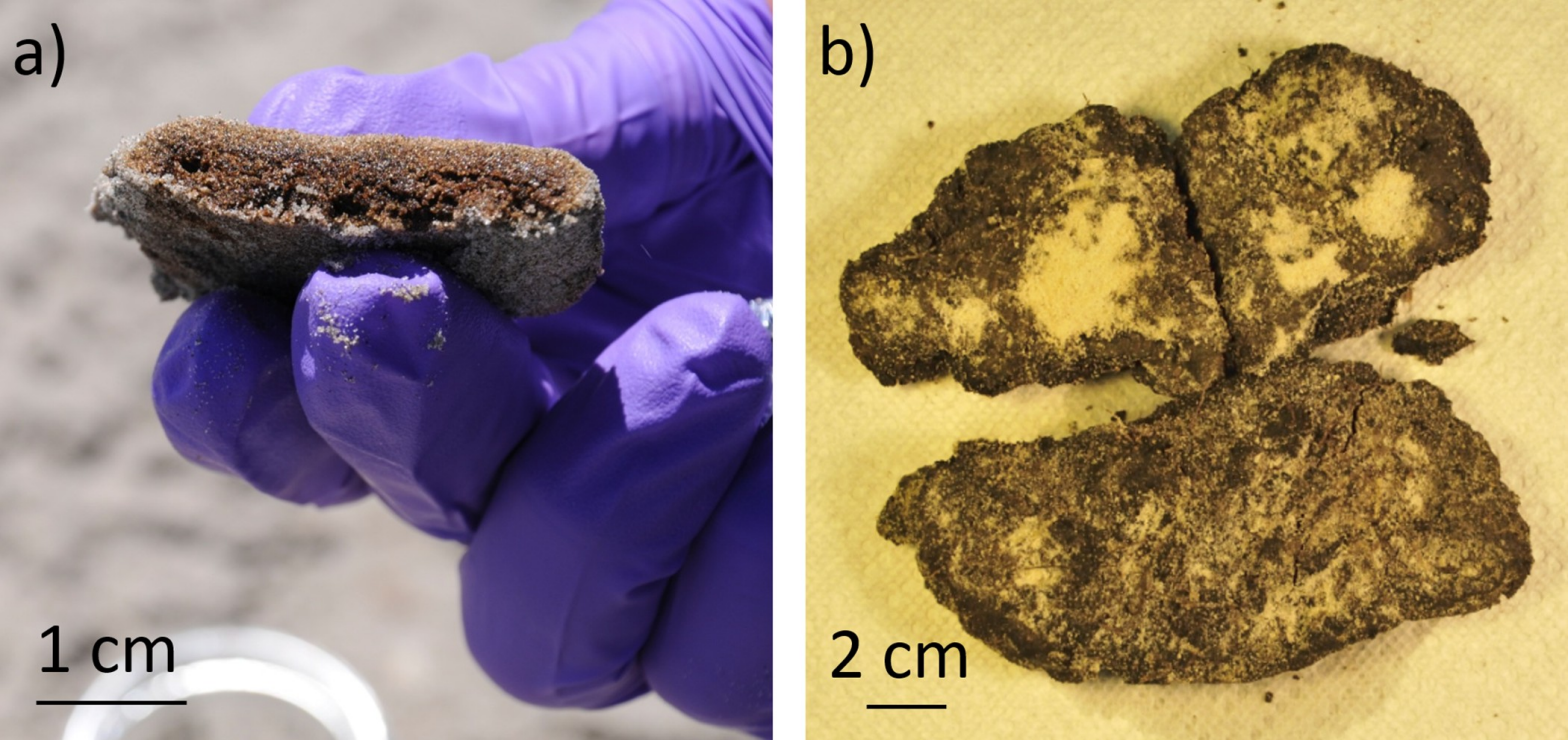
Sample images. a) Cross-section of a representative sand patty collected following the DWH spill and b) Dark-colored mat sample collected on the beach near the high tide line at Fort Morgan, AL following Hurricane *Isaac* (Aug 29, 2012).

Following Hurricane *Isaac* we also recovered several dark-colored fibrous mat pieces reported by community members as oiled material. These mats were visually similar to those previously collected in the region after Tropical Storm *Lee* [17] and on initial inspection looked as though they might contain oil residues (Fig 1 and S1 Fig). Given the potential implications and removal costs associated with misidentification of oiled debris, we sought to explore these samples beyond visual inspection. In the current research we use gas chromatography (GC), mass spectrometry (MS) and stable carbon isotopic analysis to analyze the composition and source of collected mat samples. This study aims to assess potential changes in the source of oiled sand patties collected before and after Hurricane *Isaac* and to identify the source and oil content of the unknown mat material washed ashore after the storm.

## Methods

We assessed the impact of the hurricane on the resuspension of oil residues through examination of sand patty density and chemical analysis. While the DWH spill was expected to remain the major source of oil, it was hypothesized that changes in sand patty density would be observed if the hurricane remobilized sand patties with a different degree of weathering or oil from different subsurface reservoirs. We also examined the dark-colored mat samples to determine their hydrocarbon content and source.

### Sample collection

**Fig 2:**
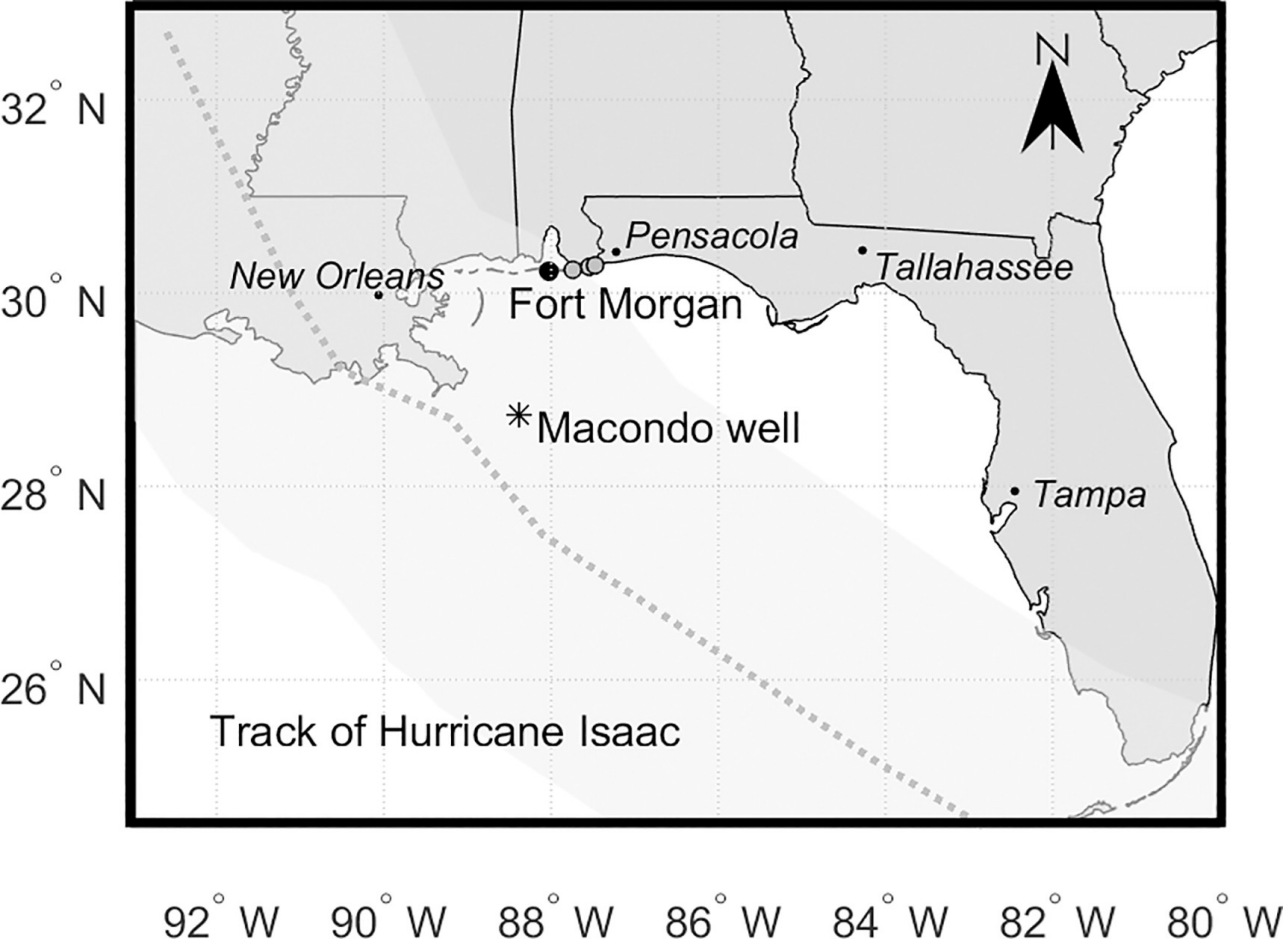
Map of the Gulf Coast region showing location of Fort Morgan, AL (black marker) and other sampling sites (grey markers), the Macondo well (the source of oil released in the DWH spill) and the track of Hurricane *Isaac* (dashed line). Masking on the track of Hurricane *Isaac* indicates the region of tropical storm-force winds extending approximately 295 km from the center of the hurricane [32]. Gulf shores, AL, Gulf State Park, AL and Perdido Beach, FL sites (from W to E) are shown in grey.

To assess the impact of Hurricane *Isaac*, we collected samples immediately following the hurricane and compared them to archived samples. For this study, potentially oiled materials were collected from Fort Morgan, AL, Gulf Shores, AL, Gulf State Park, AL, and Perdido Beach, FL (Fig 2). These sites were chosen for three reasons: 1) they were all close to the path of Hurricane *Isaac* (Fig 2); 2) we had already collected and analyzed sand patties from these sites in the months and days leading up to the hurricane, and 3) following the hurricane, community members reported submerged oil mats were being washed ashore at one of the sites (Fort Morgan, AL).

On August 30^th^, 2012 two days after the hurricane made landfall, samples were sparse or absent from all four sites visited; a two-hour survey of the beach at Fort Morgan, AL by five researchers discovered no potentially oiled material. On August 31^st^, 2012 larger sand patties (1 to 5 cm in diameter, similar to Fig 1A) were abundant at Gulf Shores but not at the other sites. On the Evening of August 31^st^, 2012 dark colored mats were reportedly washing up on the beach at Fort Morgan, and upon returning to this site the following morning, sand patties, and fibrous dark colored mat materials not previously observed at the site, were collected (Fig 1B). Large swaths of a dark mat material were observed submerged below the water line and smaller pieces near the high-tide line on the beach. Samples were collected from the mat material present higher on the beach, consisting of smaller clumps of approximately 5 x 10 cm to 25 x 30 cm, which may have broken off from the larger pieces of submerged material nearby (S2 Fig). This manuscript focuses on sand patty and mat samples collected at Fort Morgan, AL in the days before and after the hurricane (Table 1).

**Table 1:** Data for samples collected from Fort Morgan, AL.

Sample ID	Collection date	Sample type	Latitude	Longitude	% moisture	% TEM^a^	% TOC^b^
050812–01	5/8/2012	sand patty	30° 13' 28.2"^c^	88° 0' 35.7"^c^	1	28.0	N.M.^d^
050812–02	5/8/2012	sand patty	30° 13' 28.2"^c^	88° 0' 35.7"^c^	5	14.5	N.M.
050812–03	5/8/2012	sand patty	30° 13' 28.2"^c^	88° 0' 35.7"^c^	1	12.3	N.M.
050812–04	5/8/2012	sand patty	30° 13' 28.2"	88° 0' 35.7"	2	15.4	N.M.
081912–29	8/19/2012	sand patty	30° 14' 46.0"	88° 4' 33.1"	4	15.8	N.M.
082012–18	8/20/2012	sand patty	30° 13' 31.0''	88° 0' 17.6"	4	14.1	N.M.
082012–46	8/20/2012	sand patty	30° 13' 28.8"	88° 0' 31.7"	3	16.0	N.M.
082012–50	8/20/2012	sand patty	30° 13' 31.0"	88° 0' 17.5"	4	17.9	N.M.
090112–01	9/1/2012	sand patty	30° 13' 29.6''	88ᵒ 0' 30.7"	10	21.3	N.M.
090112–02	9/1/2012	sand patty	30° 13' 29.6''	88ᵒ 0' 29.5"	10	19.3	N.M.
090112–03	9/1/2012	sand patty	30° 13' 29.5''	88ᵒ 0' 30.6"	13	22.1	N.M.
090112–04	9/1/2012	mat	30° 13' 29.4''	88ᵒ 1' 10.1"	35	N.M.	4.1
090112–05	9/1/2012	mat	30° 13' 29.6''	88ᵒ 1' 9.9"	41	N.M.	9.3
090112–06	9/1/2012	mat	30° 13' 29.7''	88ᵒ 1' 9.8"	36	N.M.	7.3
090112–07	9/1/2012	mat	30° 13' 29.9''	88ᵒ 1' 10.5"	38	N.M.	16.0
090112–08	9/1/2012	mat	30° 13' 29.7''	88ᵒ 1' 9.7"	48	N.M.	5.1

^a^ total extractable material (solvent: dichloromethane) including GC and non-GC amenable hydrocarbons expressed as a percent by mass.

^b^ total organic carbon; based on measurement of material <1 mm in size, see text and S1 Text for measurement details.

^c^ approximate locations

^d^ N.M. = not measured

Sand patty samples were composite collections containing 10 to 20 individual sand patties (each 3 to 30 mm diameter) gathered from the high-tide line and within the swash zone (the region where water from breaking waves washes over the beach) along a one-kilometer section of the beach at Fort Morgan, AL. Collected sand patties were placed into combusted glass jars, and mat samples were wrapped in combusted aluminum foil before being shipped to Woods Hole, MA for analysis. Collection dates and locations of the sand patty and mat samples analyzed in this study are shown in Table 1. Archived samples from the Gulf Coast region collected from April 2011 to June 2014 were also considered in examining changes in sand patty density over time (S1 Table, S3 Fig). The methods for processing oiled sand patty samples and mat samples of unknown origin are presented separately below.

### Sand patty samples

#### Density calculations

Sand patty density was estimated for archived samples (*n* = 565) collected between April 2011 and June of 2014 from numerous Gulf Coast sites from Florida to Louisiana collected as part of a long-term study of sand patties from the DWH spill (S3 Fig). Density was calculated from percent moisture (f_H2O_) and percent extractable material (f_oil,dry_) according to Eq 1 [33]. The densities of water, sand and oil were assumed to be 1027, 2650, and 900 kg/m^3^ respectively [33]. Sample densities were calculated for all archived sand patty samples collected since the spill. Percent moisture and percent extractable material were available for 192 of the 565 samples collected. Percent moisture values ranged from 0 to 27% with 98% of samples having a moisture content between 0 and 11%. For the remaining 373 samples where percent moisture data was not available, densities were calculated using the median percent moisture (1.4%) from the smaller 192 sample data set.

$$\rho_{sandpatty}={(\frac{f_{H_{2}O}}{1027}+\frac{(1-f_{H_{2}O})\times f_{oil,dry}}{900}+\frac{1-f_{H_{2}O}-(1-f_{H_{2}O})\times f_{oil,dry}}{2650})}^{-1}$$ (1)

Non-parametric Kruskal-Wallis and post-hoc tests were used to examine changes in density with time and sampling location.

#### Sample preparation and solvent extraction

Sand patty samples were spiked with a surrogate standard solution containing *n*-dotriacontane-*d*_66_ (for total petroleum hydrocarbon, TPH, analysis) and naphthalene-*d*_8_, acenaphthene-*d*_10_, phenanthrene-*d*_10_, chrysene-*d*_12_, fluorene-*d*_10_, dibenzothiophene-*d*_8_, and fluoranthene-*d*_10_ (for polycyclic aromatic hydrocarbon, PAH, analysis), extracted three times with dichloromethane, and centrifuged to remove particulates. Solvent extracts were combined and concentrated to 1 mL by rotary evaporation before GC analyses. An *o*-terphenyl standard was added just prior to analyses to enable calculation of percent recoveries for the spiked surrogate standards.

The total extractable material (TEM) was determined by weight difference before and after sample extraction and thus includes TPHs (measured by GC) as well as non-GC amenable compounds, such as oxygenated hydrocarbons and biomass.[19,34] These oxygenated compounds are known to comprise upwards of 60% of the mass of oil from sand patties collected > 500 days after the spill [14,31].

Percent moisture was calculated on a separate sample aliquot by mass difference after samples were placed in a drying oven at 60°C overnight.

#### Gas chromatography analysis

Solvent extracts were analyzed by GC with flame ionization detection (GC-FID) to quantify the TPHs present in the GC-amenable fraction from approximately *n*-C_8_ to *n*-C_40_ [35]. Extracts were also analyzed by GC-MS to quantify parent and alkylated PAHs within samples.

TPH analyses were performed on a Hewlett-Packard 5890 series GC-FID with a split/splitless auto-injector. Samples (1 μL) were separated on a 100% dimethylpolysiloxane capillary column (DB-1MS, 30 m length, 0.25 mm I.D., 0.25 μm film thickness) with hydrogen as the carrier gas at a constant flow using methods similar to those previously described [35,36]. TPHs (which includes an unresolved complex mixture; UCM) and *n*-alkanes were quantified by integrating the total area of the FID signal and using the response factor of the *n*-dotriacontane-*d*_66_ surrogate standard. GC-FID chromatograms were also used for tier-one analysis for source determination[37]. Laboratory and method blanks were free from petroleum compounds and recoveries of surrogate standards ranged from 67 to 99%.

Analyses of PAHs within samples were performed on an Agilent 6890 Series GC coupled to an Agilent 5973 mass spectrometer. Samples (1μL) were injected in splitless mode and separated on a DB-XLB column (60 m length, 0.25 mm I.D., 0.25 μm film thickness) with helium as the carrier gas at a constant flow. We analyzed for the following parent PAHs and their C_1_ through C_3_ or C_4_ alkylated homologs using selected ion monitoring mode: naphthalene, fluorene, dibenzothiophene, phenanthrene/anthracene, fluoranthene/pyrene, and chrysene. We also measured benzo[*b*]fluoranthene, benzo[*k*]fluoranthene, benz[*a*]anthracene, benzo[*a*]pyrene, benzo[*e*]pyrene, acenaphthylene, acenaphthene, dibenz[*a*,*h*]anthracene, indeno[1,2,3-*c*,*d*]pyrene and benzo[*g*,*h*,*i*]perylene. Response factors were calculated daily by analysis of external standards containing target compounds and perdeuterated parent PAH surrogate standards. Laboratory blanks were free of analyzed petroleum compounds. Triplicate analysis indicated a precision better than ± 5% for all but two PAHs (precision better than ±10% for benzo[*k*]fluoranthene and indeno[1,2,3-*c*,*d*]pyrene), and the method detection limit was estimated to be 10 ng g^-1^ [35]. Prior to GC-MS analysis, an *o*-terphenyl standard was added to all samples. Recoveries of PAH standards averaged 79% (range 59–99%), with naphthalene-*d*_*8*_ and chrysene-*d*_*12*_ having the lowest recoveries.

### Mat samples

#### Sample preparation and solvent extraction

A six-gram aliquot of each mat sample was freeze-dried, homogenized with a spatula, spiked with the same surrogate standard solution detailed above and extracted three times with 20 mL dichloromethane:methanol (90:10). Following each extraction, samples were centrifuged to separate solvent from solids and extracts were combined. Activated copper was added to each extract to remove elemental sulfur [38]. Solvent extracts were concentrated to 1 mL by rotary evaporation before GC analyses (detailed below).

During solvent extraction of the mat samples, a flocculent formed at the surface of the supernatant and was filtered out. Low density particles and/or fine-grained materials that were trapped in the flocculent were removed and thus TEM could not be accurately determined gravimetrically. Quantification of TPHs was attempted via GC-FID analysis, but hydrocarbon concentrations in the extracts were below detection limits.

A separate aliquot from the center of each mat sample was removed with a spatula and freeze dried. Percent moisture was determined by weight difference before and after freeze-drying. Dry samples were disaggregated with a spatula and sieved into five size fractions: <150 μm, 150–250 μm, 250–500 μm, 500–1000 μm, and >1000 μm. Total organic carbon (TOC) and organic carbon isotope (δ^13^C) analyses were measured for each size fraction and bulk sediment samples by the Marine Science Institute (University of California, Santa Barbara, CA, see S1 Text).

#### Gas chromatography analysis

Chromatographic analysis was identical to methods described above for analysis of sand patty samples. However, in addition to analyzing the total extracts by GC-FID, a saturate fraction isolated by silica-gel chromatography was prepared to reduce sample complexity and allow for more accurate analysis of *n-*alkanes [39,40].

Surrogate PAH standard recoveries for mat samples averaged 77% (range 48–98%), with naphthalene-*d*_*8*_ and chrysene-*d*_*12*_ having the lowest recoveries.

## Results and discussion

We sought to address two questions: 1) Did Hurricane *Isaac* affect the weathering state, chemical composition or density of sand patties regularly washed ashore along the Gulf Coast? and 2) What was the composition of the unknown mat samples washed ashore following the storm and were they related to the DWH spill? The effect of Hurricane *Isaac* on the remobilization of oiled material was examined in several ways. First, sand patty densities were compared for all available samples collected since April 2011 (*n* = 565; S1 Table). These samples were collected at numerous Gulf Coast sites before and after Hurricane *Isaac* to explore the possibility that the high energy of the hurricane delivered denser sand patties to the beach (i.e. sand patties with a lower weight percent oil) than observed under normal wave-conditions. Second, the oil content of the mat samples collected from the beach at Fort Morgan, AL was compared to sand patty samples collected at the same site several months earlier (May 8^th^, 2012), one week prior (August 19^th^ and 20^th^, 2012), and immediately following the landfall of Hurricane *Isaac* on September 1^st^, 2012. Finally, we sought to determine the source of the dark-colored mat samples by characterizing their grain size distribution, TOC, and bulk δ^13^C composition.

### Sand patty density

The density of all samples collected since the spill was 2058 ± 209 kg/m^3^ (*n* = 565; S4 Fig) and was statistically the same (*p* = 0.073) as the small data set (*n* = 192; 2092 ± 224 kg/m^3^) where all percent moisture data were available. An independent sample *t*-test indicated that densities of our samples (*n* = 565) were slightly but significantly lower than previously reported USGS data (p = 0.011) for similar samples collected between 2010 and 2012 [33]. The USGS data set (*n* = 137) had a mean of 2107 ± 161 kg/m^3^ whereas the values for our data set were 2058 ± 209 kg/m^3^. The slightly lower average density we observed may be due to several less-dense samples (<1500 kg/m^3^; S4 Fig), differences in percent moisture content, and/or differences in sampling time/location or measurement techniques.

A Kruskal-Wallis H test was performed to determine whether observed density changes were related to time or location of sample collection. We tested samples grouped by location as well as by month and year collected. Distributions of densities were similar for all time groups analyzed (grouped by sampling month and year) and sampling locations as determined by visual inspection of boxplots. Density values were statistically different between time groups (binned by month, H_17df_ = 116, p<0.001) and sample locations (H_16df_ = 108, p<0.001). Pairwise comparisons were performed according to Dunn’s [41] procedure with a Bonferroni correction for multiple comparisons. These analyses revealed numerous statistically significant differences (all p<0.043) in density, most notably between June and July 2012 and other months from 2011 to 2014. Pairwise comparisons of sampling location data showed Gulfport, MS and Grand Isle, LA samples to be significantly different (all p<0.041; Gulfport, MS lower and Grand Isle, LA higher) from samples collected at other locations. Examination of collection dates for Gulfport, MS and Grand Isle, LA samples suggested samples from these locations drive the statistical differences observed for June and July of 2012. These data indicate no notable density changes with the passage of Hurricane *Isaac* and suggest sampling location, rather than sampling date, to be the driving factor in determining differences in sand patty density. Samples collected from different locations might have different densities from one another due to different beach sand materials that are mixed with the oil to form sand patties.

### Oil content of sand patties

**Fig 3:**
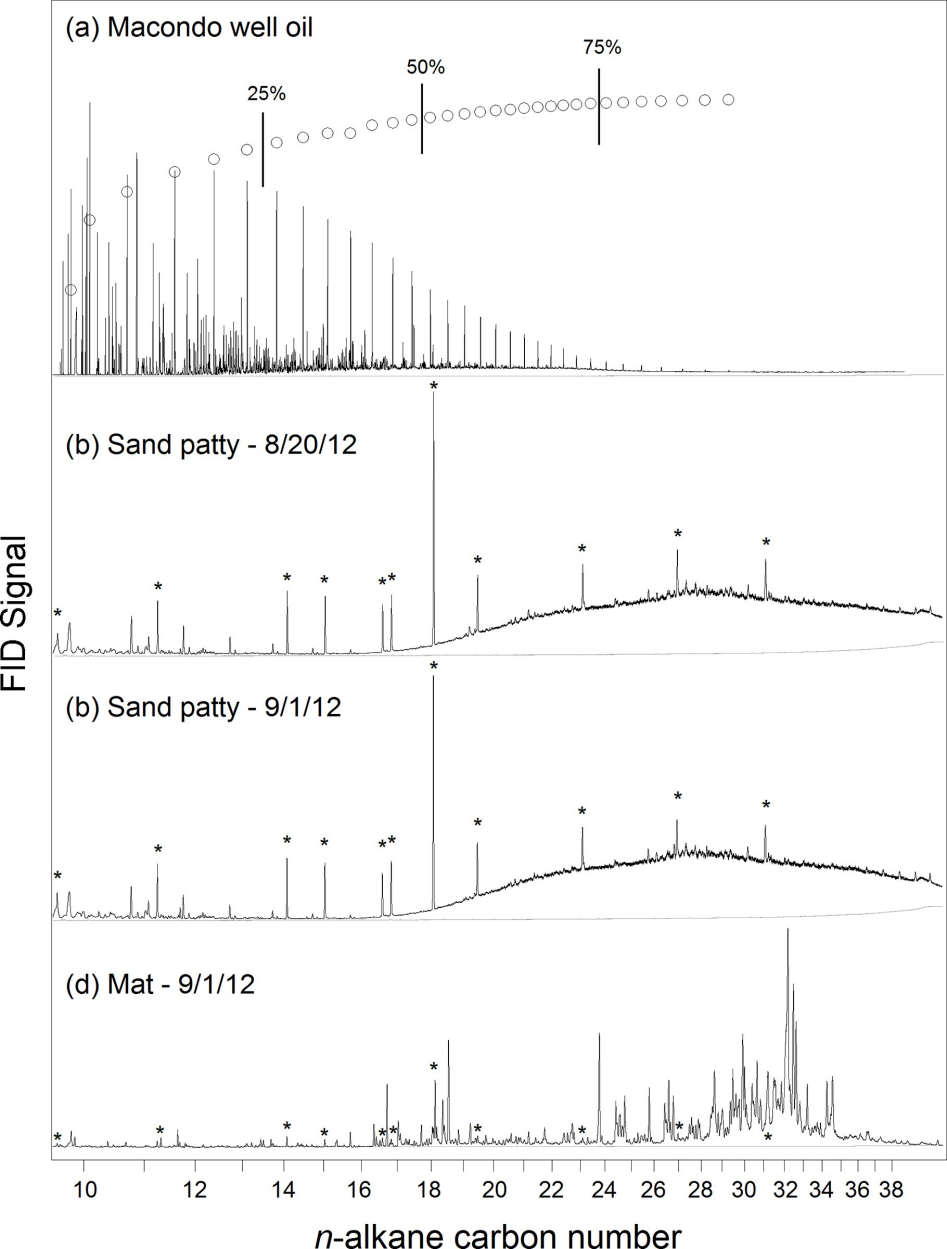
GC-FID chromatograms. Representative gas chromatograms of (a) the neat MW oil, sand patty samples collected (b) before and (c) after Hurricane *Isaac* and (d) a mat sample collected after the Hurricane *Isaac*. Simulated distillation data is also shown for the MW oil, with demarcations indicating where 25%, 50% and 75% of the GC-amenable oil mass resides. (Samples shown are the MW oil, 082012–46, 090112–02 and 090112–04 respectively; Table 1). Internal standards are indicated by asterisks (*). The grey line is the baseline of the instrument from the analysis of hexane.

The sand patties collected for this study at the four sampling sites in August 2012 (Table 1) were visually and physically consistent with sand patties tied to the DWH spill [18–20,42]. All samples met tier-1 field testing requirements used to identify DWH-related sand patties as detailed by Han and Clement (2018)[43]. Sand patty oil residues examined by GC-FID and PAH analyses were consistent with highly weathered MW oil [19,21,44]. GC-FID chromatograms of the sand patty solvent extracts contained an UCM and were absent of any *n*-alkanes (Fig 3). Biomarker analysis of samples similar in appearance and collected at study sample sites before, during and after the timeframe of this study[19,21,30,31] demonstrated these samples are consistent with residues from the DWH spill.

**Fig 4:**
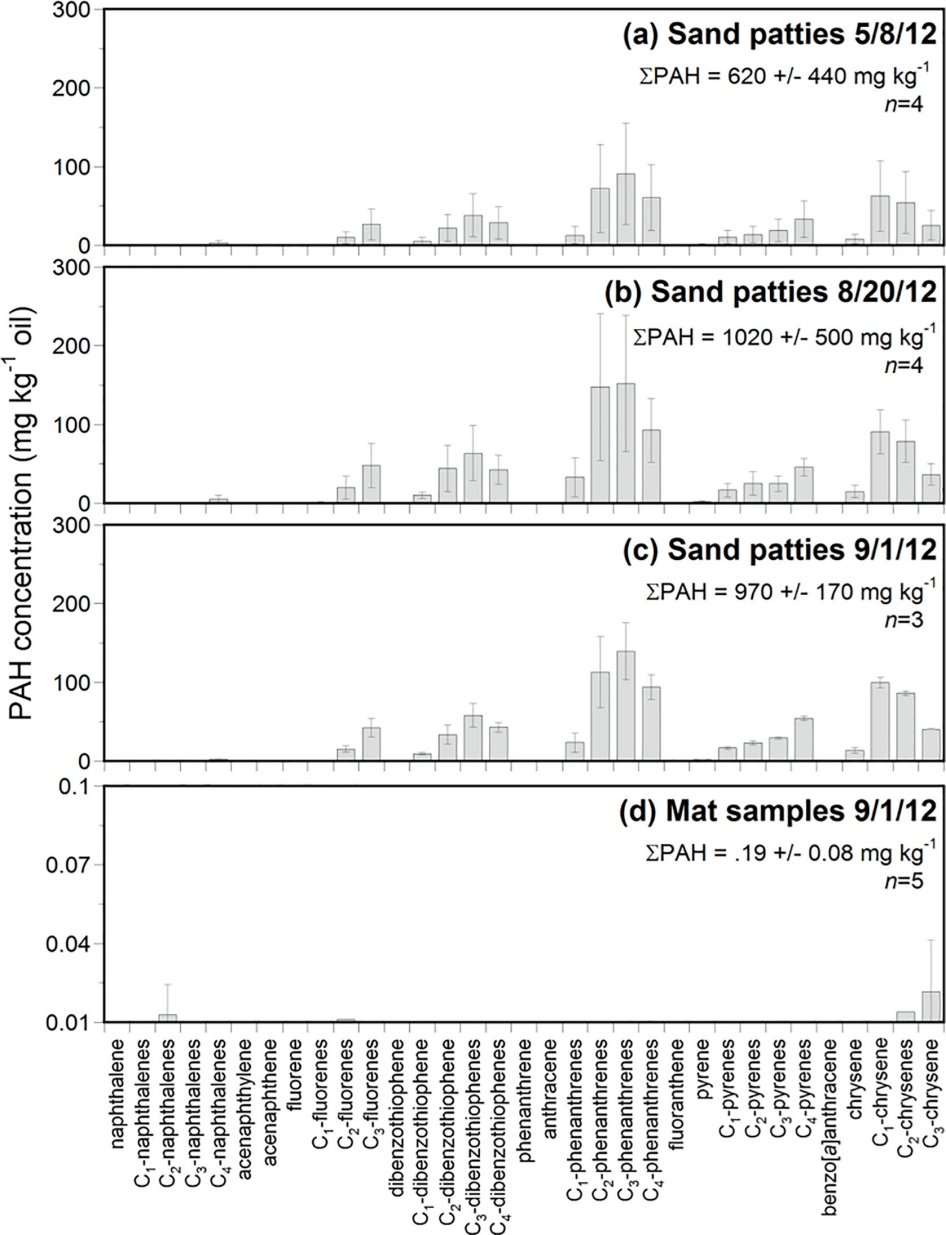
PAH content. Polycyclic aromatic hydrocarbon (PAH) content normalized to total extractable material for (a) MW oil and (b-d) average values for sand patty and mat samples collected following Hurricane *Isaac*. Collection date, total PAH (ΣPAH) content and number of samples (*n*) contributing to average values are indicated for each plot. Sample details for each date can be found in Table 1.

Oil content of all sand patties (i.e., total extractable material, containing TPH and oxygenated hydrocarbons; Table 1) averaged 18 ± 4.5% by weight with those collected before the storm having a lower oil content (17 ± 4.8%, *n =* 8) than those collected after the storm (21 ± 1.4%, *n* = 3). The UCM eluted in the *n*-C_19_ to *n*-C_38_ carbon range for all samples. Based on a simulated distillation of the MW oil, this portion of GC-amenable oil remaining in samples represented <50% of the original oil mass (Fig 3; [45]. Sand patties showed no distinct *n*-alkane peaks, indicating they had undergone extensive biodegradation [44]. The sand patties in this study are consistent with long-term weathering trends at Fort Morgan, AL seen in the years since the spill (S5 Fig) with a prominent UCM indicative of extensive biodegradation. Total PAH concentrations of analyzed sand patties (Table 1) varied from 110 to 1600 mg kg^-1^ oil (sum of 40 PAHs, see Methods) and in all samples were dominated by C_2_-C_3_ fluorenes, C_1_-C_4_ dibenezothiophenes, C_1_-C_4_ phenanthrenes/anthracenes, C_1_-C_4_ pyrenes/fluoranthenes, and C_1_-C_3_ chrysenes/benzo[*a*]anthracenes (Fig 4). The distribution of PAHs was dominated by alkylated three and four ring PAHs (e.g. phenanthrenes, dibenzothiophenes, pyrenes, and chrysenes and was similar for all sand patty samples examined in this and prior studies of DWH-derived oil residues [19,44].

Overall, there were no remarkable differences in the carbon range and PAH fingerprints of sand patties collected before (*n* = 8, May to August 2012) and after (*n* = 3, September 2012) Hurricane *Isaac*. This confirmed that there had not been a notable change in the source of oil residues (e.g. shift in submerged oil mat location) or to the degree of weathering of sand patty residues as a result of Hurricane *Isaac*.

### Oil content of mat samples

Weathered oil, as evidenced by a UCM in the GC chromatogram, was not observed in any of the dark-colored mat samples collected (Fig 3D). Also, unlike the UCM observed in sand patty samples, a multitude of compounds eluting between *n*-C_16_ and *n*-C_35_ were present. PAH concentrations were generally below the limits of detection (0.01 to 0.2 mg PAH kg^-1^ oil) (Fig 4D). Low concentrations of C_1_-, C_2_- and C_3_-chrysenes likely represent background levels due to incidental contact with oil in the Gulf region.

### Source determination of dark-colored mat samples

The *n*-alkane distribution, TOC content and δ^13^C for each of the five dark-colored mat samples were consistent with a salt marsh source. The saturate fraction of the mat samples was dominated by *n*-alkanes of odd-numbered carbon chain lengths (S6 Fig). Carbon preference indices (CPI) were calculated for the carbon range *n*-C_25_ to *n*-C_33_ according to Eq 2:

$$CPI=\frac{1}{2}\left[\frac{{(C}_{25}+C_{27}+C_{29}+C_{31})}{{(C}_{26}+C_{28}+C_{30}+C_{32})}+\frac{{(C}_{27}+C_{29}+C_{31}+C_{33})}{{(C}_{26}+C_{28}+C_{30}+C_{32})}\right]$$ (2)

The CPI values (4.8 ± 0.8) and dominance of odd chain-length *n*-alkanes is consistent with a source from higher plant waxes [46]. In contrast, petroleum derived materials have *n*-alkane distributions with no carbon preference (CPI = 1; as in [47]. For example, the calculated CPI for the MW oil is 1.2. Measured CPI values of mat samples are consistent with those calculated for salt marsh sediments [48,49].

The TOC composition of the mat samples averaged 8± 5% (Table 1; S2 Text). This is consistent with low-grade peat (<25% OC), partially decomposed plant material that is formed over time in low oxygen conditions from inundation and/or rapid burial. The largest grain size fraction (> 1 mm) contained the highest OC contents, likely as a result of plant debris (S2 Table). This OC content is within the range of other marsh sediments in the region (1–7%; [50]) and wetlands with mixed inputs of C_3_ and C_4_ vegetation (1–18%; [51]).

**Fig 5:**
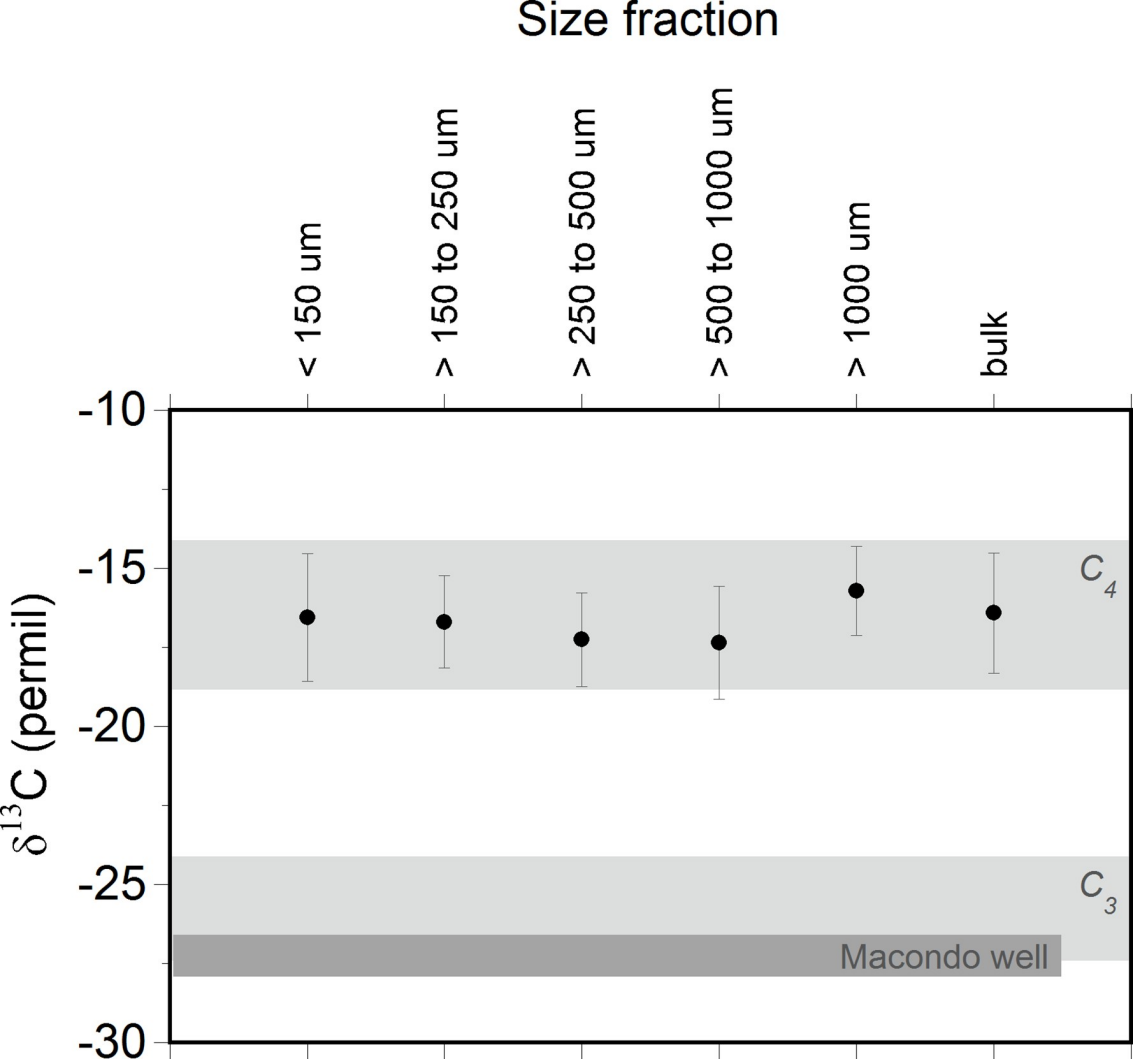
Average stable carbon isotope values (δ^13^C vs. VPDB) for the different sieved size fractions of collected mat samples. Also shown are typical values observed for C_4_ (e.g. *Spartina sp*.) and C_3_ (e.g. *Juncus*) dominated marsh sediments in the region and MW oil values [55]. The >1 mm fraction of mat samples show a slight enrichment relative to other fractions, possibly due to root debris.

The average bulk δ^13^C of the mat samples was -16.7‰ (Fig 5; S2 Table) consistent with a C_4_ dominated salt-marsh environment (-18 to -14‰; [52]. In the Gulf Coast region these marshes are commonly dominated by the grass *Spartina sp*. [52,53], a C_4_ plant with a δ^13^C of -14.8 ± 0.6‰ [52]. The more depleted sediments are the result of decomposition and subsequent removal of more labile plant components [51,52,54]. Marsh systems dominated by C_3_ plants (e.g. *Juncus*) have δ ^13^C compositions of -27 to -24‰ [51]. The measured δ^13^C of mat samples is consistent with sediments from a C_4_ dominated marsh.

Petroleum and C_3_ sources do not appear to be significant contributors to the dark-colored mat samples examined in this study. The bulk MW oil has a δ^13^C value of -27.3‰ [55] and extracts from two sand patties had δ^13^C values of -27.0 and -26.9‰ [19]. The average bulk δ^13^C of mat samples (-16.7‰) and the δ^13^C of all size fractions are inconsistent with oil contamination. If there were oil contamination we would expect more depleted δ^13^C values than those observed (Fig 5). All data are consistent with dark-colored mat samples being peat derived from a *Spartina*-dominated salt marsh that did not contain oil from the DWH spill.

## Conclusions

This study expands our understanding of the need for thorough examination of the oil content of materials washing ashore along the Gulf Coast and has implications for accurate and cost-effective shoreline cleanup, assessment and treatment endpoint determination following an oiling event.

Several conclusions can be drawn from our analyses. First, Hurricane *Isaac* did not appear to mobilize fresh or less weathered oil from submerged oil mats at the investigated field sites. Sand patties collected before and after the hurricane showed similar degrees of weathering characterized by PAH profiles, absences of *n*-alkanes and presence of a UCM.

Second, sand patties continue to be a good metric for determining beach oiling. In the current work, oiled materials were only present in sand patties suggesting their utility as an indicator of presence or absence of oil along Gulf Coast beaches.

Third, Hurricane *Isaac* brought unoiled peat samples to local beaches. Based on physical examination and GC and δ^13^C analysis, we concluded that collected mat samples were comprised of peat originating from marsh vegetation. Geochemical analysis conclusively showed these mats to be unrelated to the DWH spill by the absence of oil residues and unique δ ^13^C composition (Figs 4 and 5). The ~10‰ difference in δ ^13^C between the mat samples collected and oil from the DWH spill highlights the utility of composition of δ ^13^C for differentiating these two sample types.

Lastly, non-oiled materials can be easily confused with oiled materials. Despite their appearance, size and presence in the surf zone, all of which were similar to submerged oil mats that contained oil from the DWH spill [15,16], the mat samples examined in this study were found to contain no petroleum hydrocarbons from the DWH spill. On close inspection these peat mats can be distinguished from submerged oil mats by the presence of fibrous marsh vegetation (S1 Fig). While visual inspection can aid in minimizing false positives of oiled materials, visual analysis alone is not sufficient to determine oil content or source.

The occurrence of a storm coupled with the knowledge that DWH oil residues can re-oil coastlines can lead stakeholders to misidentify beach material resembling oiled residues as residual DWH oil. Here we present an example of such a case of misidentification. Over time and with the inevitable decrease in the quantity of oil residue in the environment from the DWH spill, differentiating samples that contain DWH-derived oil versus oil from other sources and non-oiled samples will become increasingly challenging. Through communication and education of the public following major oiling events and development of field protocols [43] it should be possible to more accurately identify heavily oiled materials enabling more efficient resource allocation and reduction of cleanup costs.

## Supporting information

S1 Text Materials and methods. (PDF)

S2 Text TOC and δ^13^C results for mat sample size fractions. (PDF)

S1 Fig Images of mat samples. Dark-colored mat samples 090112–04 to 090112–08 (a-e) collected on the beach near the high tide line at Fort Morgan, AL.
(TIF)

S2 Fig Photos of Fort Morgan, AL. Photographs of the surf zone at Fort Morgan, AL near where mat samples were collected. Photographs taken 09/01/12. Note the meter stick for scale in (b).
(TIF)

S3 Fig Map of all sampling locations of archived samples used for time-series analysis of density. Map showing sampling locations of archived samples used to examine sand patty density before and after the passing of Hurricane *Isaac*. Sample sites (from W to E): Port Fourchon, LA, Elmer’s Island, LA, Grand Isle, LA, Waveland, MS, Pass Christian, MS, Gulfport, MS, West Ship Island, MS, East Ship Island, MS, Horn Island, MS, Dauphin Island, AL, Fort Morgan, AL, Gulf Shores, AL, Gulf State Park, AL, Perdido Key, FL, Fort Pickens, FL, Pensacola Beach, FL.
(TIFF)

S4 Fig Sand patty density distribution. Distribution of densities for all sand patty samples collected by our lab since July 2011. Percent moisture and percent oil data were available for 196 (of 565) samples, for samples where no percent moisture data was available, median percent moisture (as calculated from the 196 samples) was used to calculate densities. Note the samples of low density near 1000 kg/m^3^. Samples were collected at sites shown in **S3 Fig**. USGS values are estimated from published figures [33]. Endmembers for pure oil and sand are indicated by the vertical dashed lines.
(TIFF)

S5 Fig GC-FID chromatograms showing long-term weathering trend. Four-year time series of GC-FID chromatograms of sand patty extracts collected at Fort Morgan, AL compared to Macondo well oil. Sand patty samples collected in this study were consistent with the August 2012 sample (090112–01) (c) showing a prominent UCM indicative of extensive biodegradation. Retention times have been converted to relative *n-*alkane carbon number as shown on the x-axis.
(TIF)

S6 Fig Mat sample *n*-alkane distributions. C_23_ to C_35_ *n*-alkane content determined in all mat samples collected. Odd over even dominance is present most notable in alkanes in the *n*-C_29_ to *n*-C_35_ carbon range.
(TIF)

S1 Table Archived sand patty samples collected since the DWH spill that were considered in density analysis for this study. Samples are organized by date.
(PDF)

S2 Table Grain size, carbon content and stable carbon isotopic composition (δ^13^C) of mat samples. (PDF)

## References

[1] McNutt MK, Camilli R, Crone TJ, Guthrie GD, Hsieh PA, Ryerson TB, et al Review of flow rate estimates of the *Deepwater Horizon* oil spill. Proc Natl Acad Sci. 2012;109: 20260–20267. 10.1073/pnas.1112139108 22187459 PMC3528583

[2] Michel J, Owens EH, Zengel S, Graham A, Nixon Z, Allard T, et al Extent and degree of shoreline oiling: *Deepwater Horizon* oil spill, Gulf of Mexico, USA. Chin W-C, editor. PLoS ONE. 2013;8: e65087 10.1371/journal.pone.0065087 23776444 PMC3680451

[3] National Commission on the BP *Deepwater Horizon* Oil Spill and Offshore Drilling. Deep water: the Gulf oil disaster and the future of offshore drilling—report to the President. Washington, D.C: National Commission on the BP Deepwater Horizon Oil Spill and Offshore Drilling; 2011.

[4] OSAT-2. Operational Science Advisory Team Report II: summary report for fate and effects of remnant oil in the beach environment. Gulf Coast Incident Management Team; 2011 Feb. Available: https://www.restorethegulf.gov/sites/default/files/u316/OSAT-2%20Report%20no%20ltr.pdf

[5] Wang P, Roberts TM. Distribution of surficial and buried oil contaminants across sandy beaches along NW Florida and Alabama coasts following the *Deepwater Horizon* oil spill in 2010. J Coast Res Ft Lauderdale. 2013;29: 144–155.

[6] Lin Q, Mendelssohn IA. Impacts and recovery of the *Deepwater Horizon* oil spill on vegetation structure and function of coastal salt marshes in the Northern Gulf of Mexico. Environ Sci Technol. 2012;46: 3737–3743. 10.1021/es203552p 22369124

[7] Liu Z, Liu J, Zhu Q, Wu W. The weathering of oil after the *Deepwater Horizon* oil spill: insights from the chemical composition of the oil from the sea surface, salt marshes and sediments. Environ Res Lett. 2012;7: 035302 10.1088/1748-9326/7/3/035302

[8] Mahmoudi N, Porter TM, Zimmerman AR, Fulthorpe RR, Kasozi GN, Silliman BR, et al Rapid degradation of *Deepwater Horizon* spilled oil by indigenous microbial communities in Louisiana saltmarsh sediments. Environ Sci Technol. 2013;47: 13303–13312. 10.1021/es4036072 24219093

[9] Natter M, Keevan J, Wang Y, Keimowitz AR, Okeke BC, Son A, et al Level and degradation of *Deepwater Horizon* spilled oil in coastal marsh sediments and pore-water. Environ Sci Technol. 2012;46: 5744–5755. 10.1021/es300058w 22571231

[10] Silliman BR, van de Koppel J, McCoy MW, Diller J, Kasozi GN, Earl K, et al Degradation and resilience in Louisiana salt marshes after the BP-*Deepwater Horizon* oil spill. Proc Natl Acad Sci. 2012;109: 11234–11239. 10.1073/pnas.1204922109 22733752 PMC3396483

[11] Cherry KE, Lyon BA, Marks LD, Nezat PF, Adamek R, Walsh SD, et al After the BP *Deepwater Horizon* oil spill: financial and health concerns among coastal residents and commercial fishers. Curr Psychol. 2015;34: 576–586. 10.1007/s12144-015-9372-4

[12] McCrea-Strub A, Kleisner K, Sumaila UR, Swartz W, Watson R, Zeller D, et al Potential impact of the *Deepwater Horizon* oil spill on commercial fisheries in the Gulf of Mexico. Fisheries. 2011;36: 332–336. 10.1080/03632415.2011.589334

[13] Sumaila UR, Cisneros-Montemayor AM, Dyck A, Huang L, Cheung W, Jacquet J, et al Impact of the *Deepwater Horizon* well blowout on the economics of US Gulf fisheries. Can J Fish Aquat Sci. 2012;69: 499–510. 10.1139/f2011-171

[14] Ward CP, Sharpless CM, Valentine DL, French-McCay DP, Aeppli C, White HK, et al Partial photochemical oxidation was a dominant fate of *Deepwater Horizon* surface oil. Environ Sci Technol. 2018;52: 1797–1805. 10.1021/acs.est.7b05948 29363968

[15] OSAT-3. Operational Science Advisory Team Report III: investigation of recurring residual oil in discrete shoreline areas in the eastern area of responsibility. Unified Command; 2013. Available: https://www.restorethegulf.gov/sites/default/files/u372/OSAT%20III%20Eastern%20States.pdf

[16] OSAT-3. Operational Science Advisory Team Report IV: investigation of recurring residual oil in discrete shoreline areas in Louisiana. Unified Command; 2014 Feb. Available: https://www.restorethegulf.gov/sites/default/files/u371/OSAT-3%20LA%20AOR.pdf

[17] Clement TP, Hayworth JS, Mulabagal V, John GF, Yin F. Research Brief: Is submerged *Deepwater Horizon* oil degraded offshore? Comparison of the chemical signatures of tar mat samples deposited by Tropical Storm *Lee* in September 2011 with oil mousse samples collected in June 2010 Auburn University; 2011 9 pp. 1–4.

[18] Clement TP, Hayworth JS, Mulabagal V, John GF, Yin F. Research Brief-II: Impact of Hurricane *Isaac* on Mobilizing *Deepwater Horizon* oil spill residues along Alabama’s coastline—a physiochemical characterization study Auburn University; 2012 9 pp. 1–11.

[19] Aeppli C, Carmichael CA, Nelson RK, Lemkau KL, Graham WM, Redmond MC, et al Oil weathering after the *Deepwater Horizon* disaster led to the formation of oxygenated residues. Environ Sci Technol. 2012;46: 8799–8807. 10.1021/es3015138 22809266

[20] Mulabagal V, Yin F, John GF, Hayworth JS, Clement TP. Chemical fingerprinting of petroleum biomarkers in *Deepwater Horizon* oil spill samples collected from Alabama shoreline. Mar Pollut Bull. 2013;70: 147–154. 10.1016/j.marpolbul.2013.02.026 23523118

[21] White HK, Wang CH, Williams PL, Findley DM, Thurston AM, Simister RL, et al Long-term weathering and continued oxidation of oil residues from the *Deepwater Horizon* spill. Mar Pollut Bull. 2016;113: 380–386. 10.1016/j.marpolbul.2016.10.029 27751574

[22] Dalyander PS, Long JW, Plant NG, Thompson DM. Assessing mobility and redistribution patterns of sand and oil agglomerates in the surf zone. Mar Pollut Bull. 2014;80: 200–209. 10.1016/j.marpolbul.2014.01.004 24503377

[23] Elango V, Urbano M, Lemelle KR, Pardue JH. Biodegradation of MC252 oil in oil:sand aggregates in a coastal headland beach environment. Front Microbiol. 2014;5 10.3389/fmicb.2014.00161 24782849 PMC3989593

[24] Lopatto E. Louisiana plans for gulf oil dredged by *Isaac*’s force. Bloomberg. 28 8 2012 Available: http://www.bloomberg.com/news/2012-08-28/louisiana-plans-for-gulf-oil-dredged-by-isaac-s-force.html. Accessed 2 Oct 2014.

[25] Markey E. Markey to Lubchenco letter of concern. 2012.

[26] Peeples L. Hurricane Isaac May Stir Up Oil From BP Spill. Huffington Post. 28 Aug 2012. Available: http://www.huffingtonpost.com/2012/08/28/hurricane-isaac-oil-bp-gulf-spill_n_1838064.html. Accessed 11 Jan 2014.

[27] Adelson J. About 565,000 pounds of oiled material from *Deepwater Horizon* stirred up by Hurricane Isaac Times-Picayune, NOLA.com New Orleans, LA; 17 10 2012 Available: http://www.nola.com/news/gulf-oil-spill/index.ssf/2012/10/about_565000_lbs_of_oiled_mate.html. Accessed 4 Jul 2014.

[28] International Tanker Owners Pollution Federation LTd. Disposal of oil and debris—technical information paper 9. International Tanker Owners Pollution Federation LTd; 2011.

[29] Wadsworth T. Comparison and Assessment of Waste Generated during Oil Spills (300178). International Oil Spill Conference Proceedings. 2014. pp. 1647–1658. 10.7901/2169-3358-2014.1.1647

[30] Morrison AE, Dhoonmoon C, White HK. Chemical characterization of natural and anthropogenic-derived oil residues on Gulf of Mexico beaches. Mar Pollut Bull. 2018;137: 501–508. 10.1016/j.marpolbul.2018.10.051 30503461

[31] Aeppli C, Nelson RK, Radović JR, Carmichael CA, Valentine DL, Reddy CM. Recalcitrance and degradation of petroleum biomarkers upon abiotic and biotic natural weathering of *Deepwater Horizon* oil. Environ Sci Technol. 2014;48: 6726–6734. 10.1021/es500825q 24831878

[32] Berg R. Tropical cyclone report Hurricane *Isaac* (al092012) 21 August–1 September 2012. National Hurricane Center; 2013 Jan. Report No.: al092012. Available: http://nhc.noaa.gov/data/tcr/AL092012_Isaac.pdf

[33] Plant NG, Long JW, Dalyander PS, Thompson DM, Raabe EA. Application of a hydrodynamic and sediment transport model for guidance of response efforts related to the Deepwater Horizon oil spill in the northern Gulf of Mexico along the coast of Alabama and Florida. U.S. Geological Survey; 2013. Report No.: 2012–1234. Available: http://pubs.usgs.gov/of/2012/1234/.

[34] Bostic JT, Aeppli C, Swarthout RF, Reddy CM, Ziolkowski LA. Ongoing biodegradation of *Deepwater Horizon* oil in beach sands: Insights from tracing petroleum carbon into microbial biomass. Mar Pollut Bull. 2018;126: 130–136. 10.1016/j.marpolbul.2017.10.058 29421079

[35] Lemkau KL, Peacock EE, Nelson RK, Ventura GT, Kovecses JL, Reddy CM. The M/V *Cosco Busan* spill: Source identification and short-term fate. Mar Pollut Bull. 2010;60: 2123–2129. 10.1016/j.marpolbul.2010.09.001 20888014

[36] Carmichael CA, Arey JS, Graham WM, Linn LJ, Lemkau KL, Nelson RK, et al Floating oil-covered debris from *Deepwater Horizon*: identification and application. Environ Res Lett. 2012;7: 015301 10.1088/1748-9326/7/1/015301

[37] Stout SA, Wang Z. Chemical Fingerprinting methods and factors affecting petroleum fingerprints in the environment Standard Handbook Oil Spill Environmental Forensics: Fingerprinting and Source Identification. 2nd ed. Boston, MA: Academic Press—Elsevier; 2016 pp. 61–129.

[38] Blumer M. Removal of elemental sulfur from hydrocarbon fractions. Anal Chem. 1957;29: 1039–1041. 10.1021/ac60127a014

[39] Nollet LML, editor. Chromatographic analysis of the environment. 3rd ed. Boca Raton, FL: CRC/Taylor & Franciss; 2006.

[40] Yang Z, Wang Z, Yang C, Hollebone BP, Brown C, Landriault M. Evaluation of total petroleum hydrocarbons (TPH) measurement methods for assessing oil contamination in soil. Environ Forensics. 2013;14: 193–203. 10.1080/15275922.2013.814180

[41] Dunn OJ. Multiple comparisons using rank sums. Technometrics. 1964;6 Available: http://www.tandfonline.com/doi/abs/10.1080/00401706.1964.10490181

[42] White HK, Hsing P-Y, Cho W, Shank TM, Cordes EE, Quattrini AM, et al Impact of the *Deepwater Horizon* oil spill on a deep-water coral community in the Gulf of Mexico. Proc Natl Acad Sci. 2012;109: 20303–20308. 10.1073/pnas.1118029109 22454495 PMC3528508

[43] Han Y, Clement TP. Development of a field testing protocol for identifying *Deepwater Horizon* oil spill residues trapped near Gulf of Mexico beaches. PLoS ONE. 2018;13: e0190508 10.1371/journal.pone.0190508 29329313 PMC5766100

[44] Gros J, Reddy CM, Aeppli C, Nelson RK, Carmichael CA, Arey JS. Resolving biodegradation patterns of persistent saturated hydrocarbons in weathered oil samples from the *Deepwater Horizon* disaster. Environ Sci Technol. 2014;48: 1628–1637. 10.1021/es4042836 24447243

[45] Reddy CM, Arey JS, Seewald JS, Sylva SP, Lemkau KL, Nelson RK, et al Composition and fate of gas and oil released to the water column during the *Deepwater Horizon* oil spill. Proc Natl Acad Sci. 2012;109: 20229–20234. 10.1073/pnas.1101242108 21768331 PMC3528605

[46] Reddy CM, Eglinton TI, Palić R, Benitez-Nelson BC, Stojanović G, Palić I, et al Even carbon number predominance of plant wax *n*-alkanes—a correction. Org Geochem. 2000;31: 331–336.

[47] Eglinton G, Hamilton R. Leaf epicuticular waxes. Science. 1967;156: 1322–1335. 10.1126/science.156.3780.1322 4975474

[48] Tanner BR, Uhle ME, Mora CI, Kelley JT, Schuneman PJ, Lane CS, et al Comparison of bulk and compound-specific δ13C analyses and determination of carbon sources to salt marsh sediments using *n*-alkane distributions (Maine, USA). Estuar Coast Shelf Sci. 2010;86: 283–291. 10.1016/j.ecss.2009.11.023

[49] Wang X-C, Chen RF, Berry A. Sources and preservation of organic matter in Plum Island salt marsh sediments (MA, USA): long-chain *n*-alkanes and stable carbon isotope compositions. Estuar Coast Shelf Sci. 2003;58: 917–928. 10.1016/j.ecss.2003.07.006

[50] Waterson EJ, Canuel EA. Sources of sedimentary organic matter in the Mississippi River and adjacent Gulf of Mexico as revealed by lipid biomarker and δ13C TOC analyses. Org Geochem. 2008;39: 422–439. 10.1016/j.orggeochem.2008.01.011

[51] Choi Y, Wang Y, Hsieh Y-P, Robinson L. Vegetation succession and carbon sequestration in a coastal wetland in NW Florida: evidence from carbon isotopes. Glob Biogeochem Cycles. 2001;15: 311–319.

[52] Chmura GL, Aharon P, Socki RA, Abernethy R. An inventory of 13 C abundances in coastal wetlands of Louisiana, USA: vegetation and sediments. Oecologia. 1987;74: 264–271. 10.1007/BF00379369 28312000

[53] Unified Command. MC 252 Stage III: SCAT—Shoreline treatment implementation framework for Louisiana. 2010 Sep pp. 1–95.

[54] Middelburg JJ, Nieuwenhuize J, Lubberts RK, van de Plassche O. Organic carbon isotope systematics of coastal marshes. Estuar Coast Shelf Sci. 1997;45: 681–687.

[55] Graham WM, Condon RH, Carmichael RH, D’Ambra I, Patterson HK, Linn LJ, et al Oil carbon entered the coastal planktonic food web during the *Deepwater Horizon* oil spill. Environ Res Lett. 2010;5: 045301 10.1088/1748-9326/5/4/045301

