# Supplementary material for: Hurricane Isaac brings more than oil ashore: Characteristics of beach deposits following the Deepwater Horizon spill
Source: PLoS One. 2019 Mar 18;14(3):e0213464. doi: 10.1371/journal.pone.0213464 (PMC6422254; doi:10.1371/journal.pone.0213464)
Supplement: S1 Text — (PDF) [file pone.0213464.s001.pdf]

S1 Text. Materials and Methods.

Elemental and isotopic analysis of fibrous mat samples. To determine the $\delta^{13}C$ and total organic carbon (TOC) content of the fibrous mats, samples were weighed into silver capsules, acidified with sulfurous acid to remove inorganic carbonates, and dried prior to analysis. Samples were analyzed for TOC and $\delta^{13}C$ relative to VPDB on a Finnigan Delta Plus Advantage isotope ratio mass spectrometer operated in continuous flow mode. Precision, based on 16 analyses of an acetanilide standard, were better than 2% and 0.3‰ for OC and $\delta^{13}C$ measurements respectively. Instrument accuracy was maintained using a two-point normalization and realization as described by Qui et al., (2003) [1]. Two samples were run in duplicate across the two batches of samples that were run. These replicates differed by approximately 1‰.

References

1. Qi H, Coplen TB, Geilmann H, Brand WA, Böhlke JK. Two new organic reference materials for δ13C and δ15N measurements and a new value for the δ13C of NBS 22 oil. Rapid Commun Mass Spectrom. 2003;17: 2483–2487. doi:10.1002/rcm.1219
