# Supplementary material for: Hurricane Isaac brings more than oil ashore: Characteristics of beach deposits following the Deepwater Horizon spill
Source: PLoS One. 2019 Mar 18;14(3):e0213464. doi: 10.1371/journal.pone.0213464 (PMC6422254; doi:10.1371/journal.pone.0213464)
Supplement: S2 Text — (PDF) [file pone.0213464.s002.pdf]

S2 Text. TOC content, description and $\delta^{13}C$ data for sample mat size fractions.

The grain-size distribution for all fibrous mat samples was similar and comprised primarily of particles 250-500 μm in size (57-69%; S2 Table). Visual inspection of this fraction showed that it was comprised predominantly of white root fragments, dark colored organic matter and sand. The >150 μm fraction was next most abundant (16-29%; S2 Table) and except for the absence of root material, was visually similar to the 250-500 μm fraction. These two fractions had the lowest OC content (1.5-7.2%; S2 Table) due to the large amount of sand present. General observations indicate that the grain sizes with the highest OC contents were the smallest and largest size fractions, which were the fractions that did not contain sand. The smallest fraction contained finer-grained sedimentary organic matter, while larger fractions contained plant root debris typical of marsh-derived material.
