# Supplementary figures and images for: Hurricane Isaac brings more than oil ashore: Characteristics of beach deposits following the Deepwater Horizon spill
Source: PLoS One. 2019 Mar 18;14(3):e0213464. doi: 10.1371/journal.pone.0213464 (PMC6422254; doi:10.1371/journal.pone.0213464)

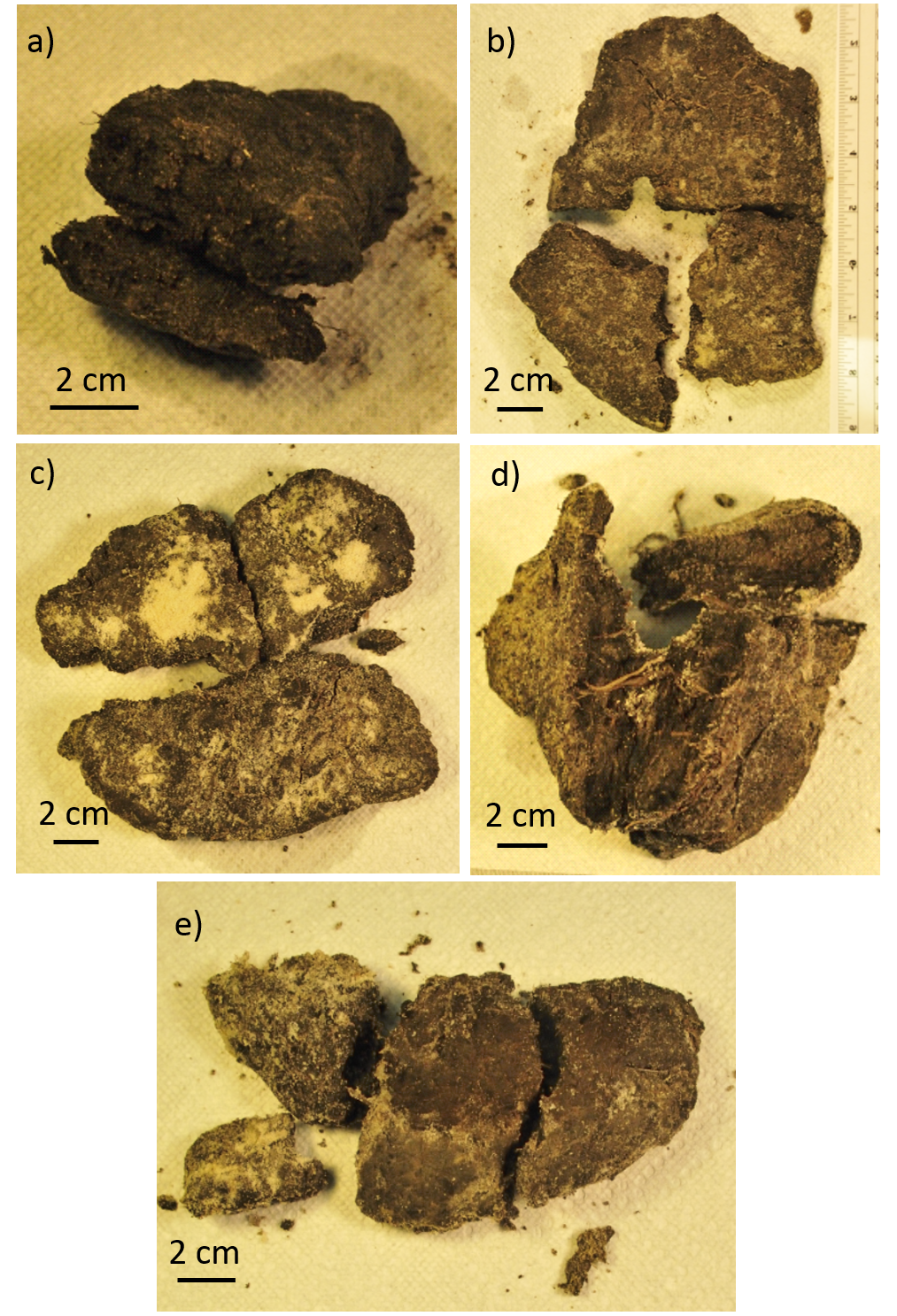

Supplement: S1 Fig — Dark-colored mat samples 090112–04 to 090112–08 (a-e) collected on the beach near the high tide line at Fort Morgan, AL. (TIF) [file pone.0213464.s003.tif]

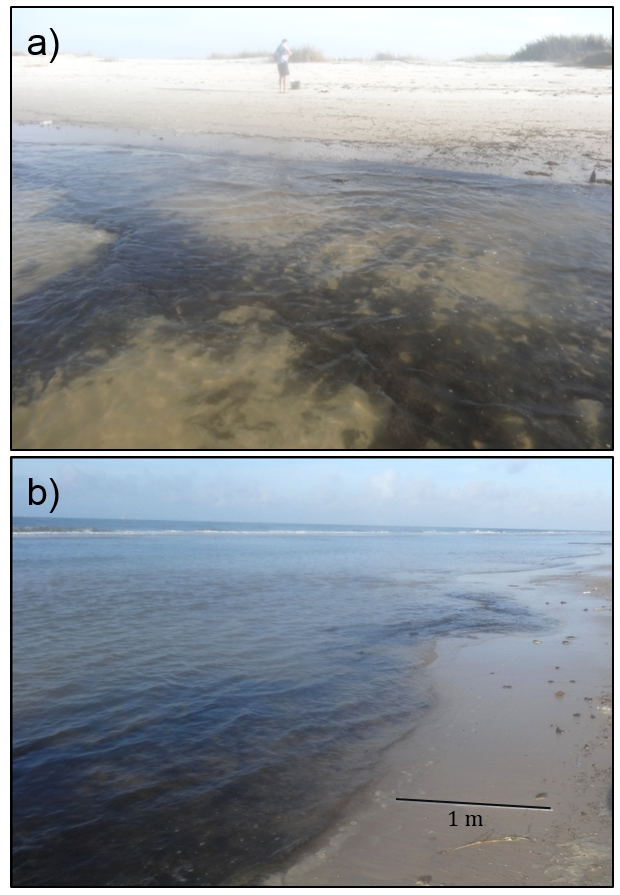

Supplement: S2 Fig — Photographs of the surf zone at Fort Morgan, AL near where mat samples were collected. Photographs taken 09/01/12. Note the meter stick for scale in (b). (TIF) [file pone.0213464.s004.tif]

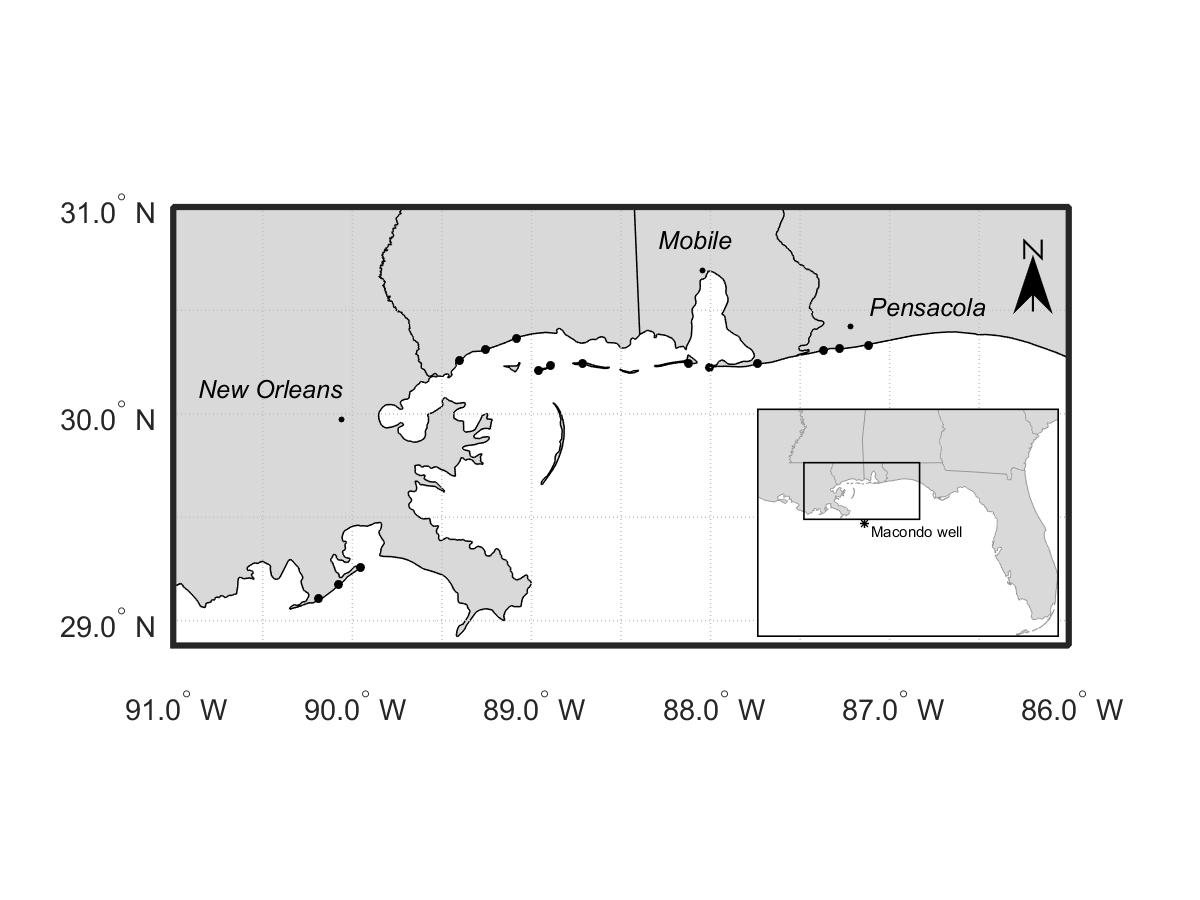

Supplement: S3 Fig — Map showing sampling locations of archived samples used to examine sand patty density before and after the passing of Hurricane Isaac. Sample sites (from W to E): Port Fourchon, LA, Elmer’s Island, LA, Grand Isle, LA, Waveland, MS, Pass Christian, MS, Gulfport, MS, West Ship Island, MS, East Ship Island, MS, Horn Island, MS, Dauphin Island, AL, Fort Morgan, AL, Gulf Shores, AL, Gulf State Park, AL, Perdido Key, FL, Fort Pickens, FL, Pensacola Beach, FL. (TIFF) [file pone.0213464.s005.tiff]

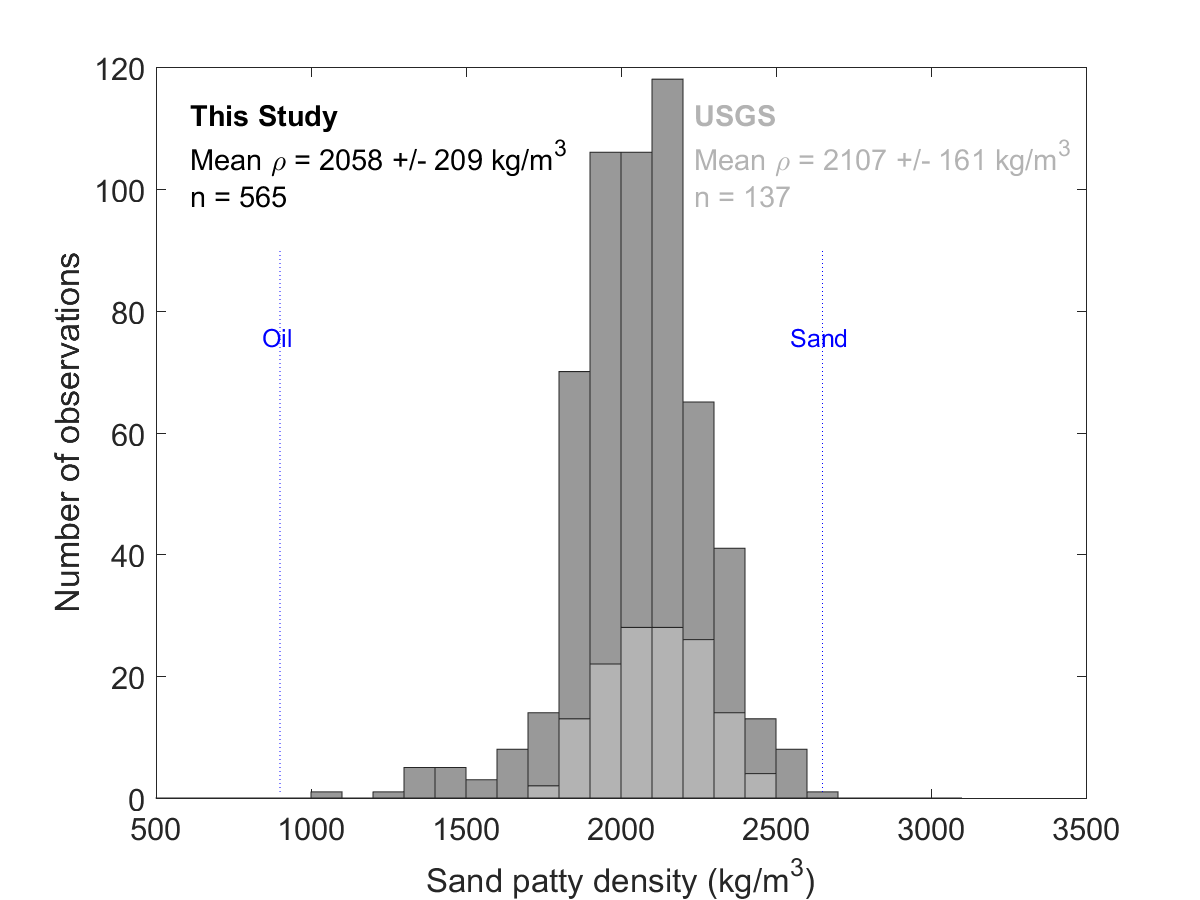

Supplement: S4 Fig — Distribution of densities for all sand patty samples collected by our lab since July 2011. Percent moisture and percent oil data were available for 196 (of 565) samples, for samples where no percent moisture data was available, median percent moisture (as calculated from the 196 samples) was used to calculate densities. Note the samples of low density near 1000 kg/m3. Samples were collected at sites shown in S3 Fig. USGS values are estimated from published figures [33]. Endmembers for pure oil and sand are indicated by the vertical dashed lines. (TIFF) [file pone.0213464.s006.tiff]

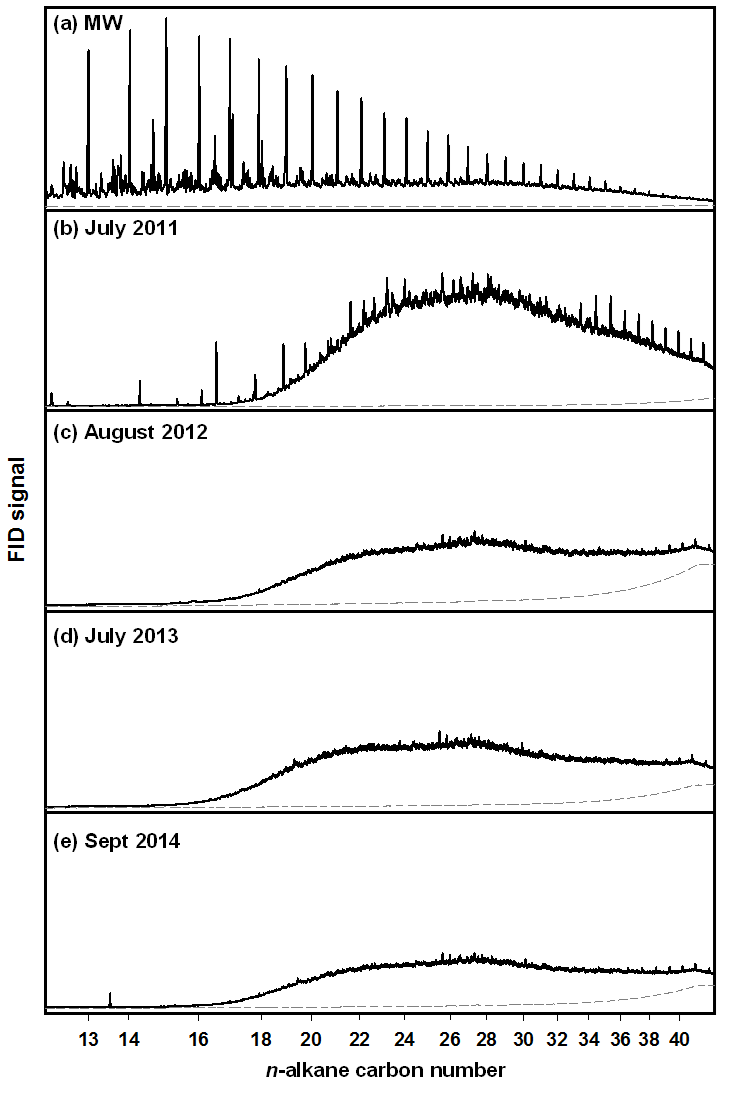

Supplement: S5 Fig — Four-year time series of GC-FID chromatograms of sand patty extracts collected at Fort Morgan, AL compared to Macondo well oil. Sand patty samples collected in this study were consistent with the August 2012 sample (090112–01) (c) showing a prominent UCM indicative of extensive biodegradation. Retention times have been converted to relative n-alkane carbon number as shown on the x-axis. (TIF) [file pone.0213464.s007.tif]

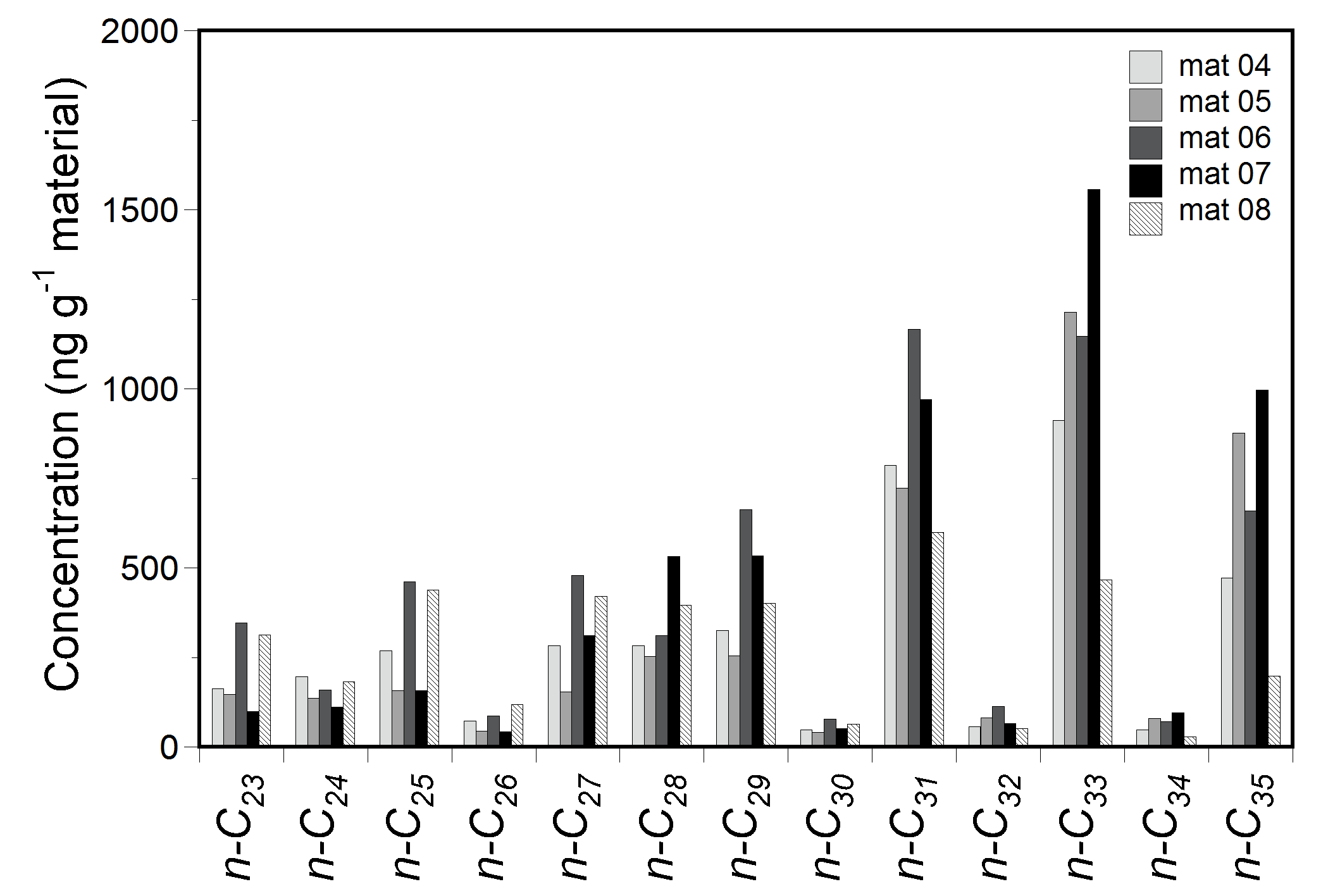

Supplement: S6 Fig — C23 to C35 n-alkane content determined in all mat samples collected. Odd over even dominance is present most notable in alkanes in the n-C29 to n-C35 carbon range. (TIF) [file pone.0213464.s008.TIF]
