# Supplementary material for: Hurricane Isaac brings more than oil ashore: Characteristics of beach deposits following the Deepwater Horizon spill
Source: PLoS One. 2019 Mar 18;14(3):e0213464. doi: 10.1371/journal.pone.0213464 (PMC6422254; doi:10.1371/journal.pone.0213464)
Supplement: S1 Table — Samples are organized by date. (PDF) [file pone.0213464.s009.pdf]

**S1 Table. Archived sand patty samples collected since the DWH spill that were considered in density analysis for this study.** Samples are organized by date.

| Sample Number | Collection Date | Sample ID | Sampling Location | Processing Lab | Fractional Oil Content | Fractional Moisture Content | Density ($kg/m^3$) |
|---|---|---|---|---|---|---|---|
| 1 | 4/3/2011 | 040311-11 | Dauphin Island, AL | Reddy[a] | 0.174 | ND | 1948 |
| 2 | 7/17/2011 | 071711-03 | Perdido Key, FL | Aeppli[b] | 0.130 | 0.003 | 2108 |
| 3 | 7/17/2011 | 071711-04 | Perdido Key, FL | Aeppli | 0.182 | 0.014 | 1932 |
| 4 | 7/17/2011 | 071711-05 | Perdido Key, FL | Aeppli | 0.148 | 0.004 | 2049 |
| 5 | 7/17/2011 | 071711-06 | Perdido Key, FL | Aeppli | 0.314 | 0.031 | 1616 |
| 6 | 7/17/2011 | 071711-07 | Perdido Key, FL | Aeppli | 0.127 | 0.003 | 2120 |
| 7 | 7/17/2011 | 071711-08 | Perdido Key, FL | Aeppli | 0.149 | 0.027 | 2000 |
| 8 | 7/17/2011 | 071711-09 | Perdido Key, FL | Aeppli | 0.200 | 0.015 | 1884 |
| 9 | 7/17/2011 | 071711-11 | Gulf Shores, AL | Aeppli | 0.149 | 0.099 | 1871 |
| 10 | 7/17/2011 | 071711-12 | Gulf Shores, AL | Aeppli | 0.141 | 0.106 | 1875 |
| 11 | 7/17/2011 | 071711-13 | Gulf Shores, AL | Aeppli | 0.142 | 0.075 | 1928 |
| 12 | 7/17/2011 | 071711-14 | Gulf Shores, AL | Aeppli | 0.133 | 0.098 | 1909 |
| 13 | 7/17/2011 | 071711-15 | Gulf Shores, AL | Aeppli | 0.126 | 0.096 | 1930 |
| 14 | 7/17/2011 | 071711-16 | Gulf Shores, AL | Aeppli | 0.173 | 0.083 | 1840 |
| 15 | 7/18/2011 | 071811-01 | Gulf Shores, AL | Aeppli | 0.128 | 0.073 | 1970 |
| 16 | 7/18/2011 | 071811-02 | Gulf Shores, AL | Aeppli | 0.164 | 0.042 | 1931 |
| 17 | 7/18/2011 | 071811-03 | Gulf Shores, AL | Aeppli | 0.215 | 0.025 | 1832 |
| 18 | 7/18/2011 | 071811-04 | Fort Morgan, AL | Aeppli | 0.147 | 0.060 | 1944 |
| 19 | 7/18/2011 | 071811-05 | Fort Morgan, AL | Aeppli | 0.242 | 0.075 | 1707 |
| 20 | 7/18/2011 | 071811-06 | Fort Morgan, AL | Aeppli | 0.127 | 0.025 | 2070 |
| 21 | 7/18/2011 | 071811-07 | Fort Morgan, AL | Aeppli | 0.139 | 0.012 | 2061 |
| 22 | 7/18/2011 | 071811-08 | Fort Morgan, AL | Aeppli | 0.121 | 0.003 | 2140 |
| 23 | 7/18/2011 | 071811-09 | Fort Morgan, AL | Aeppli | 0.151 | 0.006 | 2035 |
| 24 | 7/18/2011 | 071811-10 | Fort Morgan, AL | Aeppli | 0.123 | 0.003 | 2132 |
| 25 | 7/18/2011 | 071811-11 | Fort Morgan, AL | Aeppli | 0.158 | 0.009 | 2010 |
| 26 | 7/18/2011 | 071811-12 | Fort Morgan, AL | Aeppli | 0.137 | 0.024 | 2041 |
| 27 | 7/18/2011 | 071811-15a | Dauphin Island, AL | Aeppli | 0.120 | 0.271 | 1658 |
| 28 | 7/18/2011 | 071811-15b | Dauphin Island, AL | Reddy | 0.001 | ND | 2587 |
| 29 | 7/18/2011 | 071811-16 | Dauphin Island, AL | Reddy | 0.178 | ND | 1937 |
| 30 | 7/18/2011 | 071811-18 | Dauphin Island, AL | Reddy | 0.008 | ND | 2556 |
| 31 | 7/18/2011 | 071811-19 | Dauphin Island, AL | Reddy | 0.082 | ND | 2246 |
| 32 | 7/18/2011 | 071811-20 | Dauphin Island, AL | Reddy | 0.102 | ND | 2172 |
| 33 | 7/18/2011 | 071811-24 | Dauphin Island, AL | Reddy | 0.411 | ND | 1455 |
| 34 | 7/18/2011 | 071811-25 | Gulfport, MS | Reddy | 0.003 | ND | 2578 |
| 35 | 7/18/2011 | 071811-26 | Gulfport, MS | Reddy | 0.079 | ND | 2257 |
| 36 | 7/19/2011 | 071911-46 | Elmer's Island, LA | Aeppli | 0.109 | 0.014 | 2152 |

| | | | | | | | |
|---|---|---|---|---|---|---|---|
| 37 | 7/19/2011 | 071911-47 | Elmer's Island, LA | Aeppli | 0.110 | 0.027 | 2117 |
| 38 | 7/19/2011 | 071911-48 | Elmer's Island, LA | Aeppli | 0.041 | 0.005 | 2436 |
| 39 | 7/19/2011 | 071911-49 | Elmer's Island, LA | Aeppli | 0.097 | 0.014 | 2193 |
| 40 | 7/19/2011 | 071911-50 | Elmer's Island, LA | Aeppli | 0.053 | 0.005 | 2385 |
| 41 | 7/19/2011 | 071911-42 | Elmer's Island, LA | Reddy | 0.143 | ND | 2040 |
| 42 | 7/19/2011 | 071911-44 | Elmer's Island, LA | Reddy | 0.009 | ND | 2549 |
| 43 | 2/6/2012 | 020612-01 | Fort Pickens, FL | Reddy | 0.150 | ND | 2018 |
| 44 | 2/7/2012 | 020712-01 | Fort Pickens, FL | Reddy | 0.128 | ND | 2086 |
| 45 | 2/7/2012 | 020712-02 | Fort Pickens, FL | Reddy | 0.114 | ND | 2132 |
| 46 | 2/7/2012 | 020712-03 | Fort Pickens, FL | Reddy | 0.116 | ND | 2124 |
| 47 | 2/7/2012 | 020712-04 | Perdido Key, FL | Reddy | 0.151 | ND | 2015 |
| 48 | 2/7/2012 | 020712-05 | Perdido Key, FL | Reddy | 0.145 | ND | 2032 |
| 49 | 2/7/2012 | 020712-06 | Perdido Key, FL | Reddy | 0.208 | ND | 1859 |
| 50 | 2/7/2012 | 020712-07 | Perdido Key, FL | Reddy | 0.152 | ND | 2011 |
| 51 | 2/7/2012 | 020712-08 | Perdido Key, FL | Reddy | 0.157 | ND | 1998 |
| 52 | 2/7/2012 | 020712-09 | Perdido Key, FL | Reddy | 0.116 | ND | 2126 |
| 53 | 2/8/2012 | 020812-01 | Horn Island, MS | Reddy | 0.098 | ND | 2187 |
| 54 | 2/8/2012 | 020812-03 | Horn Island, MS | Reddy | 0.117 | ND | 2121 |
| 55 | 2/8/2012 | 020812-04 | Horn Island, MS | Reddy | 0.066 | ND | 2305 |
| 56 | 2/9/2012 | 020912-01 | West Ship Island, MS | Reddy | 0.113 | ND | 2136 |
| 57 | 2/9/2012 | 020912-02 | East Ship Island, MS | Reddy | 0.145 | ND | 2033 |
| 58 | 2/9/2012 | 020912-03 | East Ship Island, MS | Reddy | 0.117 | ND | 2121 |
| 59 | 2/9/2012 | 020912-04 | East Ship Island, MS | Reddy | 0.110 | ND | 2144 |
| 60 | 2/9/2012 | 020912-05 | East Ship Island, MS | Reddy | 0.089 | ND | 2217 |
| 61 | 2/9/2012 | 020912-06 | East Ship Island, MS | Reddy | 0.111 | ND | 2143 |
| 62 | 2/9/2012 | 020912-07 | East Ship Island, MS | Reddy | 0.165 | ND | 1974 |
| 63 | 2/9/2012 | 020912-08 | East Ship Island, MS | Reddy | 0.121 | ND | 2108 |
| 64 | 2/9/2012 | 020912-09 | East Ship Island, MS | Reddy | 0.148 | ND | 2025 |
| 65 | 3/19/2012 | 031912-01 | Perdido Key, FL | Reddy | 0.151 | ND | 2016 |
| 66 | 3/19/2012 | 031912-02 | Perdido Key, FL | Reddy | 0.144 | ND | 2035 |
| 67 | 3/19/2012 | 031912-03 | Perdido Key, FL | Reddy | 0.117 | ND | 2122 |
| 68 | 3/19/2012 | 031912-04 | Perdido Key, FL | Reddy | 0.132 | ND | 2074 |
| 69 | 3/19/2012 | 031912-05 | Gulf Shores, AL | Reddy | 0.093 | ND | 2205 |
| 70 | 3/19/2012 | 031912-06 | Gulf Shores, AL | Reddy | 0.144 | ND | 2037 |
| 71 | 3/19/2012 | 031912-09 | Gulf Shores, AL | Reddy | 0.071 | ND | 2287 |
| 72 | 3/19/2012 | 031912-10 | Gulf Shores, AL | Reddy | 0.081 | ND | 2248 |
| 73 | 3/19/2012 | 031912-11 | Gulf Shores, AL | Reddy | 0.168 | ND | 1965 |
| 74 | 3/19/2012 | 031912-12 | Gulf Shores, AL | Reddy | 0.196 | ND | 1889 |
| 75 | 3/19/2012 | 031912-13 | Gulf Shores, AL | Reddy | 0.277 | ND | 1699 |
| 76 | 3/19/2012 | 031912-14 | Gulf Shores, AL | Reddy | 0.141 | ND | 2044 |
| 77 | 3/19/2012 | 031912-15 | Gulf Shores, AL | Reddy | 0.129 | ND | 2082 |

| | | | | | | | |
|---|---|---|---|---|---|---|---|
| 78 | 3/19/2012 | 031912-16 | Gulf Shores, AL | Reddy | 0.092 | ND | 2209 |
| 79 | 3/19/2012 | 031912-17 | Gulf Shores, AL | Reddy | 0.115 | ND | 2129 |
| 80 | 3/19/2012 | 031912-18 | Gulf State Park, AL | Reddy | 0.109 | ND | 2148 |
| 81 | 3/19/2012 | 031912-19 | Gulf State Park, AL | Reddy | 0.151 | ND | 2015 |
| 82 | 3/19/2012 | 031912-20 | Gulf State Park, AL | Reddy | 0.107 | ND | 2155 |
| 83 | 3/19/2012 | 031912-21 | Gulf State Park, AL | Reddy | 0.165 | ND | 1976 |
| 84 | 3/19/2012 | 031912-22 | Gulf State Park, AL | Reddy | 0.109 | ND | 2150 |
| 85 | 3/19/2012 | 031912-23 | Gulf Shores, AL | Reddy | 0.145 | ND | 2034 |
| 86 | 3/20/2012 | 032012-01 | Dauphin Island, AL | Reddy | 0.444 | ND | 1407 |
| 87 | 3/20/2012 | 032012-02 | Dauphin Island, AL | Reddy | 0.099 | ND | 2183 |
| 88 | 3/20/2012 | 032012-03 | Dauphin Island, AL | Reddy | 0.086 | ND | 2228 |
| 89 | 3/20/2012 | 032012-04 | Dauphin Island, AL | Reddy | 0.060 | ND | 2329 |
| 90 | 3/20/2012 | 032012-05 | Dauphin Island, AL | Reddy | 0.062 | ND | 2320 |
| 91 | 3/20/2012 | 032012-06 | Dauphin Island, AL | Reddy | 0.078 | ND | 2260 |
| 92 | 3/20/2012 | 032012-07 | Dauphin Island, AL | Reddy | 0.088 | ND | 2222 |
| 93 | 3/20/2012 | 032012-08 | Pass Christian, MS | Reddy | 0.513 | ND | 1312 |
| 94 | 4/19/2012 | 041912-01 | Grand Isle, LA | Reddy | 0.191 | ND | 1902 |
| 95 | 4/19/2012 | 041912-02 | Grand Isle, LA | Reddy | 0.159 | ND | 1993 |
| 96 | 4/19/2012 | 041912-03 | Grand Isle, LA | Reddy | 0.151 | ND | 2016 |
| 97 | 4/19/2012 | 041912-04 | Grand Isle, LA | Reddy | 0.123 | ND | 2102 |
| 98 | 4/19/2012 | 041912-05 | Grand Isle, LA | Reddy | 0.112 | ND | 2138 |
| 99 | 4/19/2012 | 041912-06 | Grand Isle, LA | Reddy | 0.119 | ND | 2114 |
| 100 | 4/19/2012 | 041912-07 | Grand Isle, LA | Reddy | 0.124 | ND | 2100 |
| 101 | 4/19/2012 | 041912-08 | Grand Isle, LA | Reddy | 0.177 | ND | 1941 |
| 102 | 4/19/2012 | 041912-09 | Grand Isle, LA | Reddy | 0.192 | ND | 1901 |
| 103 | 4/19/2012 | 041912-10 | Grand Isle, LA | Reddy | 0.152 | ND | 2011 |
| 104 | 4/19/2012 | 041912-11 | Grand Isle, LA | Reddy | 0.166 | ND | 1971 |
| 105 | 4/19/2012 | 041912-12 | Grand Isle, LA | Reddy | 0.134 | ND | 2067 |
| 106 | 4/19/2012 | 041912-13 | Grand Isle, LA | Reddy | 0.183 | ND | 1923 |
| 107 | 4/19/2012 | 041912-14 | Grand Isle, LA | Reddy | 0.168 | ND | 1965 |
| 108 | 4/19/2012 | 041912-15 | Grand Isle, LA | Reddy | 0.474 | ND | 1364 |
| 109 | 4/19/2012 | 041912-16 | Grand Isle, LA | Reddy | 0.152 | ND | 2011 |
| 110 | 4/19/2012 | 041912-17 | Grand Isle, LA | Reddy | 0.152 | ND | 2014 |
| 111 | 4/19/2012 | 041912-18 | Grand Isle, LA | Reddy | 0.199 | ND | 1882 |
| 112 | 4/19/2012 | 041912-19 | Grand Isle, LA | Reddy | 0.083 | ND | 2240 |
| 113 | 4/19/2012 | 041912-20 | Grand Isle, LA | Reddy | 0.098 | ND | 2185 |
| 114 | 4/25/2012 | 042512-01 | Dauphin Island, AL | Reddy | 0.049 | ND | 2375 |
| 115 | 4/25/2012 | 042512-02 | Dauphin Island, AL | Reddy | 0.160 | ND | 1988 |
| 116 | 4/25/2012 | 042512-03 | Dauphin Island, AL | Reddy | 0.116 | ND | 2124 |
| 117 | 4/25/2012 | 042512-04 | Dauphin Island, AL | Reddy | 0.118 | ND | 2118 |
| 118 | 4/25/2012 | 042512-05 | Dauphin Island, AL | Reddy | 0.079 | ND | 2257 |

| | | | | | | | |
|---|---|---|---|---|---|---|---|
| 119 | 4/25/2012 | 042512-06 | Dauphin Island, AL | Reddy | 0.098 | ND | 2186 |
| 120 | 4/25/2012 | 042512-07 | Dauphin Island, AL | Reddy | 0.073 | ND | 2278 |
| 121 | 4/25/2012 | 042512-08 | Dauphin Island, AL | Reddy | 0.066 | ND | 2305 |
| 122 | 4/25/2012 | 042512-10 | Dauphin Island, AL | Reddy | 0.048 | ND | 2376 |
| 123 | 5/7/2012 | 050712-01 | Perdido Key, FL | Reddy | 0.071 | ND | 2284 |
| 124 | 5/7/2012 | 050712-02 | Perdido Key, FL | Reddy | 0.068 | ND | 2296 |
| 125 | 5/7/2012 | 050712-03 | Perdido Key, FL | Reddy | 0.061 | ND | 2326 |
| 126 | 5/7/2012 | 050712-04 | Perdido Key, FL | Reddy | 0.068 | ND | 2298 |
| 127 | 5/7/2012 | 050712-05 | Perdido Key, FL | Reddy | 0.087 | ND | 2227 |
| 128 | 5/7/2012 | 050712-06 | Gulf State Park, AL | Reddy | 0.096 | ND | 2195 |
| 129 | 5/7/2012 | 050712-07 | Gulf State Park, AL | Reddy | 0.152 | ND | 2011 |
| 130 | 5/7/2012 | 050712-08 | Gulf State Park, AL | Reddy | 0.137 | ND | 2059 |
| 131 | 5/7/2012 | 050712-09 | Gulf State Park, AL | Reddy | 0.220 | ND | 1829 |
| 132 | 5/7/2012 | 050712-10 | Gulf State Park, AL | Reddy | 0.186 | ND | 1916 |
| 133 | 5/7/2012 | 050712-11 | Gulf State Park, AL | Reddy | 0.151 | ND | 2017 |
| 134 | 5/7/2012 | 050712-12 | Gulf State Park, AL | Reddy | 0.149 | ND | 2020 |
| 135 | 5/7/2012 | 050712-13 | Gulf State Park, AL | Reddy | 0.190 | ND | 1906 |
| 136 | 5/7/2012 | 050712-14 | Gulf State Park, AL | Reddy | 0.171 | ND | 1958 |
| 137 | 5/7/2012 | 050712-15 | Gulf Shores, AL | Reddy | 0.168 | ND | 1967 |
| 138 | 5/7/2012 | 050712-16 | Gulf Shores, AL | Reddy | 0.115 | ND | 2130 |
| 139 | 5/7/2012 | 050712-17 | Gulf Shores, AL | Reddy | 0.159 | ND | 1990 |
| 140 | 5/7/2012 | 050712-18 | Gulf Shores, AL | Reddy | 0.163 | ND | 1980 |
| 141 | 5/7/2012 | 050712-19 | Gulf Shores, AL | Reddy | 0.144 | ND | 2036 |
| 142 | 5/7/2012 | 050712-20 | Gulf Shores, AL | Reddy | 0.130 | ND | 2079 |
| 143 | 5/7/2012 | 050712-21 | Gulf Shores, AL | Reddy | 0.137 | ND | 2056 |
| 144 | 5/7/2012 | 050712-22 | Gulf Shores, AL | Reddy | 0.163 | ND | 1979 |
| 145 | 5/8/2012 | 050812-1 | Fort Morgan, AL | Reddy | 0.280 | 0.007 | 1704 |
| 146 | 5/8/2012 | 050812-2 | Fort Morgan, AL | Reddy | 0.145 | 0.047 | 1952 |
| 147 | 5/8/2012 | 050812-3 | Fort Morgan, AL | Reddy | 0.123 | 0.007 | 2120 |
| 148 | 5/8/2012 | 050812-4 | Fort Morgan, AL | Reddy | 0.154 | 0.023 | 1986 |
| 149 | 5/8/2012 | 050812-06 | Dauphin Island, AL | Reddy | 0.248 | ND | 1763 |
| 150 | 5/8/2012 | 050812-22 | Dauphin Island, AL | Reddy | 0.042 | ND | 2403 |
| 151 | 5/8/2012 | 050812-23 | Dauphin Island, AL | Reddy | 0.046 | ND | 2384 |
| 152 | 5/8/2012 | 050812-25 | Dauphin Island, AL | Reddy | 0.088 | ND | 2223 |
| 153 | 5/8/2012 | 050812-26a | Dauphin Island, AL | Reddy | 0.197 | ND | 1886 |
| 154 | 5/9/2012 | 050912-06 | Waveland, MS | Reddy | 0.409 | ND | 1459 |
| 155 | 5/9/2012 | 050912-07 | Waveland, MS | Reddy | 0.192 | ND | 1900 |
| 156 | 5/9/2012 | 050912-08 | Waveland, MS | Reddy | 0.157 | ND | 1998 |
| 157 | 6/12/2012 | 061212-4 | Dauphin Island, AL | White[c] | 0.095 | 0.003 | 2230 |
| 158 | 6/12/2012 | 061212-5 | Dauphin Island, AL | White | 0.066 | 0.049 | 2196 |
| 159 | 6/12/2012 | 061212-6 | Gulf Shores, AL | White | 0.092 | 0.014 | 2208 |

| | | | | | | | |
|---|---|---|---|---|---|---|---|
| 160 | 6/12/2012 | 061212-7 | Gulf Shores, AL | White | 0.082 | 0.025 | 2211 |
| 161 | 6/12/2012 | 061212-8 | Perdido Key, FL | White | 0.056 | 0.001 | 2388 |
| 162 | 6/12/2012 | 061212-13 | Gulf Shores, AL | White | 0.107 | 0.002 | 2188 |
| 163 | 6/12/2012 | 061212-14 | Gulf Shores, AL | White | 0.096 | 0.090 | 1994 |
| 164 | 6/12/2012 | 061212-15 | Gulf Shores, AL | White | 0.103 | 0.003 | 2201 |
| 165 | 6/12/2012 | 061212-16 | Perdido Key, FL | White | 0.047 | 0.006 | 2408 |
| 166 | 6/12/2012 | 061212-17 | Perdido Key, FL | White | 0.055 | 0.018 | 2333 |
| 167 | 6/12/2012 | 061212-18 | Perdido Key, FL | White | 0.012 | 0.001 | 2585 |
| 168 | 6/12/2012 | 061212-19 | Perdido Key, FL | White | 0.042 | 0.002 | 2447 |
| 169 | 6/13/2012 | 061312-2 | Waveland, MS | White | 0.075 | 0.003 | 2306 |
| 170 | 6/13/2012 | 061312-6 | Waveland, MS | White | 0.070 | 0.009 | 2303 |
| 171 | 6/13/2012 | 061312-11 | Waveland, MS | White | 0.307 | 0.016 | 1634 |
| 172 | 6/13/2012 | 061312-12 | West Ship Island, MS | White | 0.070 | 0.001 | 2328 |
| 173 | 6/13/2012 | 061312-12 | Waveland, MS | White | 0.245 | 0.012 | 1773 |
| 174 | 6/13/2012 | 061312-13 | Waveland, MS | White | 0.094 | 0.004 | 2228 |
| 175 | 6/13/2012 | 061312-14 | West Ship Island, MS | White | 0.049 | 0.022 | 2343 |
| 176 | 6/13/2012 | 061312-21 | West Ship Island, MS | White | 0.067 | 0.009 | 2313 |
| 177 | 6/14/2012 | 061412-1 | Elmer's Island, LA | White | 0.032 | 0.002 | 2487 |
| 178 | 6/14/2012 | 061412-2 | Grand Isle, LA | White | 0.081 | 0.008 | 2264 |
| 179 | 6/14/2012 | 061412-3 | Elmer's Island, LA | White | 0.039 | 0.003 | 2455 |
| 180 | 6/14/2012 | 061412-5 | Grand Isle, LA | White | 0.081 | 0.008 | 2265 |
| 181 | 6/14/2012 | 061412-8 | Grand Isle, LA | White | 0.090 | 0.023 | 2189 |
| 182 | 6/14/2012 | 061412-10 | Grand Isle, LA | White | 0.081 | 0.067 | 2098 |
| 183 | 6/14/2012 | 061412-10 | Elmer's Island, LA | White | 0.037 | 0.003 | 2464 |
| 184 | 6/14/2012 | 061412-11 | Grand Isle, LA | White | 0.151 | 0.093 | 1840 |
| 185 | 6/14/2012 | 061412-12 | Grand Isle, LA | White | 0.014 | 0.058 | 2369 |
| 186 | 6/14/2012 | 061412-13 | Grand Isle, LA | White | 0.175 | 0.071 | 1825 |
| 187 | 6/14/2012 | 061412-14 | Grand Isle, LA | White | 0.121 | 0.004 | 2134 |
| 188 | 6/14/2012 | 061412-16 | Grand Isle, LA | White | 0.151 | 0.008 | 2031 |
| 189 | 6/14/2012 | 061412-17 | Grand Isle, LA | White | 0.014 | 0.071 | 2327 |
| 190 | 6/14/2012 | 061412-19 | Grand Isle, LA | White | 0.175 | 0.016 | 1941 |
| 191 | 6/21/2012 | 062112-02 | Waveland, MS | Reddy | 0.121 | ND | 2108 |
| 192 | 6/21/2012 | 062112-03 | Waveland, MS | Reddy | 0.080 | ND | 2253 |
| 193 | 6/26/2012 | 062612-1 | Pensacola Beach, FL | White | 0.073 | 0.025 | 2241 |
| 194 | 6/26/2012 | 062612-2 | Pensacola Beach, FL | White | 0.011 | 0.001 | 2589 |
| 195 | 6/26/2012 | 062612-3 | Pensacola Beach, FL | White | 0.125 | 0.025 | 2067 |
| 196 | 6/26/2012 | 062612-4 | Pensacola Beach, FL | White | 0.036 | 0.003 | 2467 |
| 197 | 6/26/2012 | 062612-5 | Pensacola Beach, FL | White | 0.051 | 0.004 | 2396 |
| 198 | 6/26/2012 | 062612-12 | Dauphin Island, AL | White | 0.046 | 0.004 | 2421 |
| 199 | 6/26/2012 | 062612-13 | Dauphin Island, AL | White | 0.077 | 0.035 | 2202 |
| 200 | 6/26/2012 | 062612-15 | Dauphin Island, AL | White | 0.073 | 0.002 | 2313 |

| | | | | | | | |
|---|---|---|---|---|---|---|---|
| 201 | 6/26/2012 | 062612-19 | Dauphin Island, AL | White | 0.057 | 0.029 | 2293 |
| 202 | 6/26/2012 | 062612-20 | Perdido Key, FL | White | 0.066 | 0.001 | 2345 |
| 203 | 6/26/2012 | 062612-22 | Gulf Shores, AL | White | 0.050 | 0.024 | 2333 |
| 204 | 6/27/2012 | 062712-20 | Waveland, MS | White | 0.069 | 0.012 | 2297 |
| 205 | 6/27/2012 | 062712-26 | West Ship Island, MS | White | 0.111 | 0.000 | 2179 |
| 206 | 6/27/2012 | 062712-27 | West Ship Island, MS | White | 0.055 | 0.005 | 2377 |
| 207 | 6/27/2012 | 062712-30 | West Ship Island, MS | White | 0.076 | 0.008 | 2282 |
| 208 | 6/28/2012 | 062812-4 | Port Fourchon, LA | White | 0.084 | 0.004 | 2263 |
| 209 | 6/28/2012 | 062812-7 | Port Fourchon, LA | White | 0.086 | 0.007 | 2249 |
| 210 | 6/28/2012 | 062812-8 | Port Fourchon, LA | White | 0.174 | 0.001 | 1978 |
| 211 | 6/28/2012 | 062812-24 | Elmer's Island, LA | White | 0.106 | 0.002 | 2193 |
| 212 | 6/28/2012 | 062812-31 | Grand Isle, LA | White | 0.126 | 0.063 | 1973 |
| 213 | 7/17/2012 | 071712-5 | Fort Pickens, FL | White | 0.080 | 0.013 | 2251 |
| 214 | 7/17/2012 | 071712-6 | Pensacola Beach, FL | White | 0.032 | 0.001 | 2490 |
| 215 | 7/17/2012 | 071712-6 | Fort Pickens, FL | White | 0.047 | 0.000 | 2429 |
| 216 | 7/17/2012 | 071712-9 | Pensacola Beach, FL | White | 0.012 | 0.002 | 2581 |
| 217 | 7/17/2012 | 071712-21 | Dauphin Island, AL | White | 0.063 | 0.003 | 2350 |
| 218 | 7/17/2012 | 071712-24 | Gulf Shores, AL | White | 0.039 | 0.043 | 2320 |
| 219 | 7/17/2012 | 071712-25 | Gulf Shores, AL | White | 0.036 | 0.027 | 2381 |
| 220 | 7/17/2012 | 071712-25 | Dauphin Island, AL | White | 0.216 | 0.003 | 1859 |
| 221 | 7/17/2012 | 071712-26 | Dauphin Island, AL | White | 0.198 | 0.087 | 1740 |
| 222 | 7/17/2012 | 071712-27 | Dauphin Island, AL | White | 0.188 | 0.009 | 1920 |
| 223 | 7/18/2012 | 071812-2 | Gulfport, MS | White | 0.178 | 0.019 | 1927 |
| 224 | 7/18/2012 | 071812-3 | Gulfport, MS | White | 0.166 | 0.012 | 1976 |
| 225 | 7/18/2012 | 071812-4 | Gulfport, MS | White | 0.136 | 0.038 | 2000 |
| 226 | 7/18/2012 | 071812-7 | Gulfport, MS | White | 0.123 | 0.009 | 2114 |
| 227 | 7/18/2012 | 071812-11 | Gulfport, MS | White | 0.102 | 0.006 | 2193 |
| 228 | 7/18/2012 | 071812-21 | Gulfport, MS | White | 0.069 | 0.003 | 2327 |
| 229 | 7/18/2012 | 071812-42 | West Ship Island, MS | White | 0.085 | 0.005 | 2259 |
| 230 | 7/18/2012 | 071812-44 | West Ship Island, MS | White | 0.098 | 0.004 | 2214 |
| 231 | 7/19/2012 | 071912-28 | Elmer's Island, LA | White | 0.102 | 0.003 | 2205 |
| 232 | 7/19/2012 | 071912-29 | Elmer's Island, LA | White | 0.067 | 0.076 | 2120 |
| 233 | 7/19/2012 | 071912-30 | Elmer's Island, LA | White | 0.040 | 0.078 | 2208 |
| 234 | 7/19/2012 | 071912-32 | Grand Isle, LA | White | 0.101 | 0.006 | 2200 |
| 235 | 7/19/2012 | 071912-33 | Grand Isle, LA | White | 0.054 | 0.064 | 2197 |
| 236 | 7/19/2012 | 071912-34 | Grand Isle, LA | White | 0.060 | 0.007 | 2350 |
| 237 | 7/19/2012 | 071912-37 | Grand Isle, LA | White | 0.004 | 0.002 | 2622 |
| 238 | 7/19/2012 | 071912-38 | Grand Isle, LA | White | 0.039 | 0.002 | 2458 |
| 239 | 7/19/2012 | 071912-332 | Grand Isle, LA | White | 0.066 | 0.001 | 2342 |
| 240 | 8/19/2012 | 081912-29 | Fort Morgan, AL | Reddy | 0.158 | 0.038 | 1939 |
| 241 | 8/19/2012 | 081912-25 | Fort Morgan, AL | Reddy | 0.336 | ND | 1583 |

| | | | | | | | |
|---|---|---|---|---|---|---|---|
| 242 | 8/19/2012 | 081912-27 | Fort Morgan, AL | Reddy | 0.141 | ND | 2047 |
| 243 | 8/19/2012 | 081912-28 | Dauphin Island, AL | Reddy | 0.484 | ND | 1350 |
| 244 | 8/19/2012 | 081912-30 | Gulf Shores, AL | Reddy | 0.194 | ND | 1896 |
| 245 | 8/19/2012 | 081912-31 | Gulf Shores, AL | Reddy | 0.205 | ND | 1866 |
| 246 | 8/19/2012 | 081912-32 | Gulf Shores, AL | Reddy | 0.218 | ND | 1832 |
| 247 | 8/19/2012 | 081912-33 | Gulf Shores, AL | Reddy | 0.191 | ND | 1901 |
| 248 | 8/19/2012 | 081912-34 | Dauphin Island, AL | Reddy | 0.111 | ND | 2142 |
| 249 | 8/19/2012 | 081912-35 | Gulf Shores, AL | Reddy | 0.219 | ND | 1831 |
| 250 | 8/19/2012 | 081912-37 | Fort Morgan, AL | Reddy | 0.129 | ND | 2082 |
| 251 | 8/19/2012 | 081912-40 | Dauphin Island, AL | Reddy | 0.464 | ND | 1378 |
| 252 | 8/19/2012 | 081912-41 | Dauphin Island, AL | Reddy | 0.330 | ND | 1594 |
| 253 | 8/19/2012 | 081912-42 | Dauphin Island, AL | Reddy | 0.470 | ND | 1369 |
| 254 | 8/19/2012 | 081912-43 | Dauphin Island, AL | Reddy | 0.357 | ND | 1544 |
| 255 | 8/20/2012 | 082012-18 | Fort Morgan, AL | Reddy | 0.141 | 0.042 | 1978 |
| 256 | 8/20/2012 | 082012-46 | Fort Morgan, AL | Reddy | 0.160 | 0.034 | 1941 |
| 257 | 8/20/2012 | 082012-50 | Fort Morgan, AL | Reddy | 0.179 | 0.036 | 1888 |
| 258 | 8/20/2012 | 082012-01 | Dauphin Island, AL | Reddy | 0.074 | ND | 2275 |
| 259 | 8/20/2012 | 082012-02 | Dauphin Island, AL | Reddy | 0.064 | ND | 2314 |
| 260 | 8/20/2012 | 082012-03 | Fort Morgan, AL | Reddy | 0.108 | ND | 2152 |
| 261 | 8/20/2012 | 082012-04 | Fort Morgan, AL | Reddy | 0.106 | ND | 2160 |
| 262 | 8/20/2012 | 082012-05 | Fort Morgan, AL | Reddy | 0.137 | ND | 2057 |
| 263 | 8/20/2012 | 082012-06 | Fort Morgan, AL | Reddy | 0.124 | ND | 2098 |
| 264 | 8/20/2012 | 082012-07 | Fort Morgan, AL | Reddy | 0.143 | ND | 2039 |
| 265 | 8/20/2012 | 082012-08 | Fort Morgan, AL | Reddy | 0.130 | ND | 2080 |
| 266 | 8/20/2012 | 082012-09 | Fort Morgan, AL | Reddy | 0.162 | ND | 1984 |
| 267 | 8/20/2012 | 082012-10 | Fort Morgan, AL | Reddy | 0.100 | ND | 2179 |
| 268 | 8/20/2012 | 082012-11 | Fort Morgan, AL | Reddy | 0.087 | ND | 2227 |
| 269 | 8/20/2012 | 082012-12 | Dauphin Island, AL | Reddy | 0.117 | ND | 2123 |
| 270 | 8/20/2012 | 082012-13 | Fort Morgan, AL | Reddy | 0.133 | ND | 2071 |
| 271 | 8/20/2012 | 082012-14 | Dauphin Island, AL | Reddy | 0.067 | ND | 2300 |
| 272 | 8/20/2012 | 082012-15 | Dauphin Island, AL | Reddy | 0.052 | ND | 2359 |
| 273 | 8/20/2012 | 082012-17 | Dauphin Island, AL | Reddy | 0.065 | ND | 2309 |
| 274 | 8/20/2012 | 082012-18 | Fort Morgan, AL | Reddy | 0.128 | ND | 2088 |
| 275 | 8/20/2012 | 082012-19 | Fort Morgan, AL | Reddy | 0.124 | ND | 2098 |
| 276 | 8/20/2012 | 082012-20 | Dauphin Island, AL | Reddy | 0.138 | ND | 2054 |
| 277 | 8/20/2012 | 082012-21 | Dauphin Island, AL | Reddy | 0.064 | ND | 2312 |
| 278 | 8/20/2012 | 082012-22 | Fort Morgan, AL | Reddy | 0.154 | ND | 2005 |
| 279 | 8/20/2012 | 082012-23 | Fort Morgan, AL | Reddy | 0.072 | ND | 2282 |
| 280 | 8/20/2012 | 082012-24 | Fort Morgan, AL | Reddy | 0.193 | ND | 1898 |
| 281 | 8/20/2012 | 082012-25 | Fort Morgan, AL | Reddy | 0.097 | ND | 2192 |
| 282 | 8/20/2012 | 082012-27 | Fort Morgan, AL | Reddy | 0.174 | ND | 1950 |

| | | | | | | | |
|---|---|---|---|---|---|---|---|
| 283 | 8/20/2012 | 082012-28 | Fort Morgan, AL | Reddy | 0.149 | ND | 2021 |
| 284 | 8/20/2012 | 082012-29 | Fort Morgan, AL | Reddy | 0.120 | ND | 2111 |
| 285 | 8/20/2012 | 082012-30 | Fort Morgan, AL | Reddy | 0.117 | ND | 2121 |
| 286 | 8/20/2012 | 082012-31 | Fort Morgan, AL | Reddy | 0.173 | ND | 1952 |
| 287 | 8/20/2012 | 082012-32 | Fort Morgan, AL | Reddy | 0.177 | ND | 1940 |
| 288 | 8/20/2012 | 082012-33 | Fort Morgan, AL | Reddy | 0.178 | ND | 1937 |
| 289 | 8/20/2012 | 082012-34 | Fort Morgan, AL | Reddy | 0.150 | ND | 2019 |
| 290 | 8/20/2012 | 082012-35 | Fort Morgan, AL | Reddy | 0.180 | ND | 1932 |
| 291 | 8/20/2012 | 082012-36 | Fort Morgan, AL | Reddy | 0.180 | ND | 1932 |
| 292 | 8/20/2012 | 082012-37 | Fort Morgan, AL | Reddy | 0.105 | ND | 2162 |
| 293 | 8/20/2012 | 082012-38 | Fort Morgan, AL | Reddy | 0.122 | ND | 2104 |
| 294 | 8/20/2012 | 082012-39 | Fort Morgan, AL | Reddy | 0.141 | ND | 2047 |
| 295 | 8/20/2012 | 082012-40 | Fort Morgan, AL | Reddy | 0.140 | ND | 2050 |
| 296 | 8/20/2012 | 082012-41 | Fort Morgan, AL | Reddy | 0.137 | ND | 2057 |
| 297 | 8/20/2012 | 082012-42 | Fort Morgan, AL | Reddy | 0.097 | ND | 2191 |
| 298 | 8/20/2012 | 082012-43 | Fort Morgan, AL | Reddy | 0.151 | ND | 2015 |
| 299 | 8/20/2012 | 082012-44 | Fort Morgan, AL | Reddy | 0.153 | ND | 2010 |
| 300 | 8/20/2012 | 082012-45 | Fort Morgan, AL | Reddy | 0.125 | ND | 2096 |
| 301 | 8/20/2012 | 082012-46 | Fort Morgan, AL | Reddy | 0.549 | ND | 1268 |
| 302 | 8/20/2012 | 082012-47 | Fort Morgan, AL | Reddy | 0.103 | ND | 2171 |
| 303 | 8/20/2012 | 082012-50 | Fort Morgan, AL | Reddy | 0.154 | ND | 2006 |
| 304 | 8/20/2012 | 082012-51 | Fort Morgan, AL | Reddy | 0.182 | ND | 1926 |
| 305 | 8/20/2012 | 082012-52 | Fort Morgan, AL | Reddy | 0.183 | ND | 1924 |
| 306 | 8/20/2012 | 082012-53 | Fort Morgan, AL | Reddy | 0.134 | ND | 2066 |
| 307 | 8/20/2012 | 082012-54 | Fort Morgan, AL | Reddy | 0.113 | ND | 2136 |
| 308 | 8/20/2012 | 082012-56 | Fort Morgan, AL | Reddy | 0.132 | ND | 2075 |
| 309 | 8/20/2012 | 082012-57 | Fort Morgan, AL | Reddy | 0.165 | ND | 1974 |
| 310 | 8/20/2012 | 082012-58 | Fort Morgan, AL | Reddy | 0.125 | ND | 2097 |
| 311 | 8/20/2012 | 082012-59 | Fort Morgan, AL | Reddy | 0.177 | ND | 1940 |
| 312 | 8/20/2012 | 082012-60 | Fort Morgan, AL | Reddy | 0.150 | ND | 2018 |
| 313 | 8/20/2012 | 082012-61 | Fort Morgan, AL | Reddy | 0.186 | ND | 1916 |
| 314 | 8/20/2012 | 082012-62 | Fort Morgan, AL | Reddy | 0.117 | ND | 2121 |
| 315 | 8/20/2012 | 082012-66 | Fort Morgan, AL | Reddy | 0.141 | ND | 2046 |
| 316 | 8/20/2012 | 082012-67 | Fort Morgan, AL | Reddy | 0.143 | ND | 2039 |
| 317 | 8/20/2012 | 082012-68 | Fort Morgan, AL | Reddy | 0.129 | ND | 2082 |
| 318 | 9/1/2012 | 090112-1 | Fort Morgan, AL | Reddy | 0.213 | 0.101 | 1683 |
| 319 | 9/1/2012 | 090112-2 | Fort Morgan, AL | Reddy | 0.193 | 0.100 | 1728 |
| 320 | 9/1/2012 | 090112-3 | Fort Morgan, AL | Reddy | 0.221 | 0.133 | 1616 |
| 321 | 12/2/2012 | 120212-02-mds | Gulf Shores, AL | Reddy | 0.191 | ND | 1904 |
| 322 | 12/2/2012 | 120212-04-cc | Gulf Shores, AL | Reddy | 0.194 | ND | 1894 |
| 323 | 12/3/2012 | 0120312-5 | Fort Morgan, AL | Reddy | 0.118 | ND | 2117 |

| | | | | | | | |
|---|---|---|---|---|---|---|---|
| 324 | 12/3/2012 | 0120312-6 | Fort Morgan, AL | Reddy | 0.288 | ND | 1676 |
| 325 | 12/3/2012 | 0120312-7 | Fort Morgan, AL | Reddy | 0.202 | ND | 1875 |
| 326 | 12/3/2012 | 0120312-8 | Fort Morgan, AL | Reddy | 0.185 | ND | 1917 |
| 327 | 12/3/2012 | 0120312-9 | Fort Morgan, AL | Reddy | 0.100 | ND | 2179 |
| 328 | 12/3/2012 | 120312-05 | Fort Morgan, AL | Reddy | 0.118 | ND | 2117 |
| 329 | 12/3/2012 | 120312-07 | Fort Morgan, AL | Reddy | 0.202 | ND | 1875 |
| 330 | 12/3/2012 | 120312-10 | Fort Morgan, AL | Reddy | 0.117 | ND | 2121 |
| 331 | 12/4/2012 | 120412-01 Comp. | Grand Isle, LA | Reddy | 0.119 | ND | 2116 |
| 332 | 12/4/2012 | 120412-02 Comp. | Grand Isle, LA | Reddy | 0.147 | ND | 2027 |
| 333 | 12/4/2012 | 120412-03 Comp. | Grand Isle, LA | Reddy | 0.166 | ND | 1972 |
| 334 | 4/25/2013 | 042513-12 | Pensacola Beach, FL | White | 0.154 | 0.039 | 1949 |
| 335 | 6/12/2013 | 061213-10 | Fort Morgan, AL | Reddy | 0.201 | ND | 1875 |
| 336 | 6/12/2013 | 061213-11 | Fort Morgan, AL | Reddy | 0.203 | ND | 1871 |
| 337 | 6/12/2013 | 061213-12 | Fort Morgan, AL | Reddy | 0.120 | ND | 2111 |
| 338 | 6/12/2013 | 061213-13 | Fort Morgan, AL | Reddy | 0.212 | ND | 1849 |
| 339 | 6/12/2013 | 061213-14 | Fort Morgan, AL | Reddy | 0.194 | ND | 1894 |
| 340 | 6/12/2013 | 061213-15 | Fort Morgan, AL | Reddy | 0.188 | ND | 1910 |
| 341 | 6/12/2013 | 061213-17 | Fort Morgan, AL | Reddy | 0.197 | ND | 1888 |
| 342 | 6/12/2013 | 061213-01 | Perdido Key, FL | Reddy | 0.140 | ND | 2047 |
| 343 | 6/12/2013 | 061213-02 | Perdido Key, FL | Reddy | 0.160 | ND | 1988 |
| 344 | 6/12/2013 | 061213-04 | Perdido Key, FL | Reddy | 0.179 | ND | 1936 |
| 345 | 6/12/2013 | 061213-05 | Perdido Key, FL | Reddy | 0.191 | ND | 1901 |
| 346 | 6/12/2013 | 061213-06 | Perdido Key, FL | Reddy | 0.180 | ND | 1932 |
| 347 | 6/12/2013 | 061213-07 | Perdido Key, FL | Reddy | 0.174 | ND | 1948 |
| 348 | 6/12/2013 | 061213-08 | Fort Morgan, AL | Reddy | 0.196 | ND | 1889 |
| 349 | 6/12/2013 | 061213-09 | Fort Morgan, AL | Reddy | 0.197 | ND | 1886 |
| 350 | 6/12/2013 | 061213-10 | Fort Morgan, AL | Reddy | 0.201 | ND | 1875 |
| 351 | 6/12/2013 | 061213-11 | Fort Morgan, AL | Reddy | 0.203 | ND | 1871 |
| 352 | 6/12/2013 | 061213-13 | Fort Morgan, AL | Reddy | 0.212 | ND | 1849 |
| 353 | 6/12/2013 | 061213-14 | Fort Morgan, AL | Reddy | 0.194 | ND | 1894 |
| 354 | 6/12/2013 | 061213-15 | Fort Morgan, AL | Reddy | 0.188 | ND | 1910 |
| 355 | 6/12/2013 | 061213-16 | Fort Morgan, AL | Reddy | 0.192 | ND | 1900 |
| 356 | 6/12/2013 | 061213-17 | Fort Morgan, AL | Reddy | 0.197 | ND | 1888 |
| 357 | 6/12/2013 | 061213-18 | Gulf Shores, AL | Reddy | 0.224 | ND | 1818 |
| 358 | 6/12/2013 | 061213-19 | Gulf Shores, AL | Reddy | 0.196 | ND | 1889 |
| 359 | 6/12/2013 | 061213-20 | Gulf Shores, AL | Reddy | 0.170 | ND | 1960 |
| 360 | 6/12/2013 | 061213-21 | Gulf Shores, AL | Reddy | 0.153 | ND | 2008 |
| 361 | 6/12/2013 | 061213-22 | Gulf Shores, AL | Reddy | 0.169 | ND | 1962 |
| 362 | 6/14/2013 | 061413-04 | Elmer's Island, LA | Reddy | 0.136 | ND | 2061 |
| 363 | 6/14/2013 | 061413-05 | Elmer's Island, LA | Reddy | 0.175 | ND | 1945 |
| 364 | 7/9/2013 | 070913-1 | Fort Morgan, AL | White | 0.105 | 0.089 | 1973 |

| | | | | | | | |
|---|---|---|---|---|---|---|---|
| 365 | 7/9/2013 | 070913-2 | Fort Morgan, AL | White | 0.235 | 0.051 | 1724 |
| 366 | 7/9/2013 | 070913-12 | Fort Pickens, FL | White | 0.105 | 0.020 | 2145 |
| 367 | 7/9/2013 | 070913-15 | Fort Pickens, FL | White | 0.113 | 0.000 | 2173 |
| 368 | 7/9/2013 | 070913-25 | Perdido Key, FL | White | 0.128 | 0.000 | 2122 |
| 369 | 7/9/2013 | 070913-27 | Gulf Shores, AL | White | 0.110 | 0.009 | 2157 |
| 370 | 7/9/2013 | 070913-28 | Gulf Shores, AL | White | 0.056 | 0.000 | 2391 |
| 371 | 7/9/2013 | 070913-28 | Dauphin Island, AL | White | 0.134 | 0.059 | 1958 |
| 372 | 7/9/2013 | 070913-31 | Dauphin Island, AL | White | 0.113 | 0.010 | 2145 |
| 373 | 7/9/2013 | 070913-33 | Dauphin Island, AL | White | 0.130 | 0.110 | 1858 |
| 374 | 7/9/2013 | 070913-41 | Fort Morgan, AL | Reddy | 0.105 | 0.090 | 1968 |
| 375 | 7/9/2013 | 070913-05 Comp. | Fort Pickens, FL | Reddy | 0.094 | ND | 2202 |
| 376 | 7/9/2013 | 070913-07 | Fort Pickens, FL | Reddy | 0.212 | ND | 1849 |
| 377 | 7/9/2013 | 070913-08 Comp. | Fort Pickens, FL | Reddy | 0.254 | ND | 1749 |
| 378 | 7/9/2013 | 070913-09 | Fort Pickens, FL | Reddy | 0.193 | ND | 1897 |
| 379 | 7/9/2013 | 070913-10 | Fort Pickens, FL | Reddy | 0.201 | ND | 1877 |
| 380 | 7/9/2013 | 070913-13 Comp. | Fort Morgan, AL | Reddy | 0.207 | ND | 1860 |
| 381 | 7/9/2013 | 070913-14 | Fort Morgan, AL | Reddy | 0.182 | ND | 1926 |
| 382 | 7/9/2013 | 070913-15 | Fort Morgan, AL | Reddy | 0.253 | ND | 1750 |
| 383 | 7/9/2013 | 070913-16 | Fort Morgan, AL | Reddy | 0.211 | ND | 1851 |
| 384 | 7/9/2013 | 070913-17 Comp. | Fort Morgan, AL | Reddy | 0.197 | ND | 1887 |
| 385 | 7/9/2013 | 070913-18 | Fort Morgan, AL | Reddy | 0.311 | ND | 1629 |
| 386 | 7/9/2013 | 070913-19 Comp. | Dauphin Island, AL | Reddy | 0.226 | ND | 1814 |
| 387 | 7/9/2013 | 070913-20 Comp. | Dauphin Island, AL | Reddy | 0.199 | ND | 1881 |
| 388 | 7/9/2013 | 070913-21 | Dauphin Island, AL | Reddy | 0.235 | ND | 1791 |
| 389 | 7/9/2013 | 070913-22 | Dauphin Island, AL | Reddy | 0.206 | ND | 1865 |
| 390 | 7/9/2013 | 070913-22 | Dauphin Island, AL | Reddy | 0.092 | ND | 2208 |
| 391 | 7/9/2013 | 070913-23 | Dauphin Island, AL | Reddy | 0.205 | ND | 1865 |
| 392 | 7/9/2013 | 070913-24 | Dauphin Island, AL | Reddy | 0.151 | ND | 2016 |
| 393 | 7/9/2013 | 070913-B01 | Perdido Key, FL | Reddy | 0.177 | ND | 1941 |
| 394 | 7/9/2013 | 070913-B02 Comp. | Perdido Key, FL | Reddy | 0.162 | ND | 1983 |
| 395 | 7/9/2013 | 070913-B07 Comp. | Perdido Key, FL | Reddy | 0.234 | ND | 1794 |
| 396 | 7/9/2013 | 070913-B08 Comp. | Gulf Shores, AL | Reddy | 0.099 | ND | 2185 |
| 397 | 7/9/2013 | 070913-B10 Comp. | Fort Morgan, AL | Reddy | 0.193 | ND | 1897 |
| 398 | 7/10/2013 | 071013-23 | Gulfport, MS | White | 0.152 | 0.000 | 2045 |
| 399 | 7/10/2013 | 071013-25 | Gulfport, MS | White | 0.150 | 0.002 | 2046 |
| 400 | 7/10/2013 | 071013-51 | West Ship Island, MS | White | 0.104 | 0.000 | 2204 |
| 401 | 7/10/2013 | 071013-52 | West Ship Island, MS | White | 0.116 | 0.002 | 2158 |
| 402 | 7/10/2013 | 071013-55 | West Ship Island, MS | White | 0.121 | 0.082 | 1943 |
| 403 | 7/10/2013 | 071013- 01 Comp. | West Ship Island, MS | Reddy | 0.085 | ND | 2234 |
| 404 | 7/10/2013 | 071013-02 Comp. | West Ship Island, MS | Reddy | 0.055 | ND | 2348 |
| 405 | 7/10/2013 | 071013-03 | West Ship Island, MS | Reddy | 0.092 | ND | 2209 |

| | | | | | | | |
|---|---|---|---|---|---|---|---|
| 406 | 7/10/2013 | 071013-04 | West Ship Island, MS | Reddy | 0.142 | ND | 2043 |
| 407 | 7/10/2013 | 071013-05 | West Ship Island, MS | Reddy | 0.099 | ND | 2182 |
| 408 | 7/10/2013 | 071013-08 | West Ship Island, MS | Reddy | 0.117 | ND | 2121 |
| 409 | 7/10/2013 | 071013-09 Comp. | West Ship Island, MS | Reddy | 0.114 | ND | 2131 |
| 410 | 7/10/2013 | 071013-11 | West Ship Island, MS | Reddy | 0.001 | ND | 2588 |
| 411 | 7/10/2013 | 071013-13 Comp. | West Ship Island, MS | Reddy | 0.102 | ND | 2172 |
| 412 | 7/10/2013 | 071013-14 Comp. | West Ship Island, MS | Reddy | 0.101 | ND | 2178 |
| 413 | 7/10/2013 | 071013-15 Comp. | West Ship Island, MS | Reddy | 0.063 | ND | 2318 |
| 414 | 7/10/2013 | 071013-21 | West Ship Island, MS | Reddy | 0.127 | ND | 2088 |
| 415 | 7/10/2013 | 071013-24 Comp. | West Ship Island, MS | Reddy | 0.115 | ND | 2127 |
| 416 | 7/10/2013 | 071013-25 | West Ship Island, MS | Reddy | 0.154 | ND | 2006 |
| 417 | 7/10/2013 | 071013-26 Comp. | West Ship Island, MS | Reddy | 0.182 | ND | 1927 |
| 418 | 7/10/2013 | 071013-27 | West Ship Island, MS | Reddy | 0.106 | ND | 2159 |
| 419 | 7/10/2013 | 071013-28 Comp. | West Ship Island, MS | Reddy | 0.206 | ND | 1864 |
| 420 | 7/10/2013 | 071013-31 Comp. | West Ship Island, MS | Reddy | 0.167 | ND | 1969 |
| 421 | 7/11/2013 | 071113-40 | Grand Isle, LA | White | 0.067 | 0.108 | 2037 |
| 422 | 7/11/2013 | 071113-46 | Grand Isle, LA | White | 0.199 | 0.000 | 1911 |
| 423 | 7/11/2013 | 071113-48 | Grand Isle, LA | White | 0.111 | 0.061 | 2021 |
| 424 | 7/11/2013 | 071113- 08 | Grand Isle, LA | Reddy | 0.775 | ND | 1048 |
| 425 | 7/11/2013 | 071113-01 Comp. | Grand Isle, LA | Reddy | 0.113 | ND | 2137 |
| 426 | 7/11/2013 | 071113-03 Comp. | Grand Isle, LA | Reddy | 0.075 | ND | 2270 |
| 427 | 7/11/2013 | 071113-09 | Grand Isle, LA | Reddy | 0.114 | ND | 2133 |
| 428 | 7/11/2013 | 071113-10 Comp. | Grand Isle, LA | Reddy | 0.108 | ND | 2151 |
| 429 | 7/11/2013 | 071113-14 Comp. | Grand Isle, LA | Reddy | 0.075 | ND | 2270 |
| 430 | 7/11/2013 | 071113-15 Comp. | Grand Isle, LA | Reddy | 0.087 | ND | 2227 |
| 431 | 7/11/2013 | 071113-16 | Grand Isle, LA | Reddy | 0.130 | ND | 2081 |
| 432 | 7/11/2013 | 071113-B02 | Grand Isle, LA | Reddy | 0.179 | ND | 1934 |
| 433 | 7/11/2013 | 071113-B03 Comp. | Grand Isle, LA | Reddy | 0.107 | ND | 2154 |
| 434 | 7/11/2013 | 071113-B06 Comp. | Grand Isle, LA | Reddy | 0.185 | ND | 1919 |
| 435 | 11/21/2013 | 0112113-7 | Fort Morgan, AL | White | 0.141 | 0.014 | 2044 |
| 436 | 11/21/2013 | 0112113-8 | Fort Morgan, AL | Reddy | 0.124 | 0.010 | 2109 |
| 437 | 11/21/2013 | 0112113-9 | Fort Morgan, AL | Reddy | 0.228 | 0.005 | 1825 |
| 438 | 11/21/2013 | 0112113-11 | Fort Morgan, AL | Reddy | 0.142 | 0.015 | 2041 |
| 439 | 11/21/2013 | 0112113-12 | Fort Morgan, AL | Reddy | 0.156 | 0.027 | 1969 |
| 440 | 11/21/2013 | 0112113-13 | Fort Morgan, AL | Reddy | 0.200 | 0.031 | 1843 |
| 441 | 11/21/2013 | 112113-01 | Dauphin Island, AL | Reddy | 0.092 | ND | 2208 |
| 442 | 11/21/2013 | 112113-02 | Dauphin Island, AL | Reddy | 0.108 | ND | 2154 |
| 443 | 11/22/2013 | 112213-01 | Gulf State Park, AL | Reddy | 0.108 | ND | 2152 |
| 444 | 11/22/2013 | 112213-02 | Gulf State Park, AL | Reddy | 0.273 | ND | 1706 |
| 445 | 11/22/2013 | 112213-03 | Gulf State Park, AL | Reddy | 0.132 | ND | 2074 |
| 446 | 11/22/2013 | 112213-04 | Gulf State Park, AL | Reddy | 0.185 | ND | 1920 |

| | | | | | | | |
|---|---|---|---|---|---|---|---|
| 447 | 11/22/2013 | 112213-06 | Fort Pickens, FL | Reddy | 0.098 | ND | 2187 |
| 448 | 11/22/2013 | 112213-07 | Fort Pickens, FL | Reddy | 0.099 | ND | 2182 |
| 449 | 11/22/2013 | 112213-07B | Fort Pickens, FL | Reddy | 0.112 | ND | 2140 |
| 450 | 11/22/2013 | 112213-08 | Fort Pickens, FL | Reddy | 0.197 | ND | 1888 |
| 451 | 11/22/2013 | 112213-09 | Fort Pickens, FL | Reddy | 0.140 | ND | 2049 |
| 452 | 11/23/2013 | 112313-01 | Fort Pickens, FL | Reddy | 0.078 | ND | 2258 |
| 453 | 11/23/2013 | 112313-02 | Fort Pickens, FL | Reddy | 0.105 | ND | 2163 |
| 454 | 11/23/2013 | 112313-03 | Fort Pickens, FL | Reddy | 0.129 | ND | 2084 |
| 455 | 11/23/2013 | 112313-04 | Fort Pickens, FL | Reddy | 0.111 | ND | 2143 |
| 456 | 11/23/2013 | 112313-05 | Fort Pickens, FL | Reddy | 0.139 | ND | 2051 |
| 457 | 11/23/2013 | 112313-06 | Fort Pickens, FL | Reddy | 0.113 | ND | 2134 |
| 458 | 11/23/2013 | 112313-07 | Fort Pickens, FL | Reddy | 0.190 | ND | 1905 |
| 459 | 11/23/2013 | 112313-08 | Fort Pickens, FL | Reddy | 0.199 | ND | 1881 |
| 460 | 11/23/2013 | 112313-09b | Fort Pickens, FL | Reddy | 0.164 | ND | 1978 |
| 461 | 11/23/2013 | 112313-10 | Fort Pickens, FL | Reddy | 0.166 | ND | 1970 |
| 462 | 11/23/2013 | 112313-11 | Fort Pickens, FL | Reddy | 0.446 | ND | 1404 |
| 463 | 11/23/2013 | 112313-12 | Fort Pickens, FL | Reddy | 0.171 | ND | 1957 |
| 464 | 1/25/2014 | 012514-3 | Fort Morgan, AL | White | 0.090 | 0.024 | 2184 |
| 465 | 1/25/2014 | 012514-26 | Perdido Key, FL | White | 0.190 | 0.019 | 1893 |
| 466 | 1/25/2014 | 012514-30 | Gulf Shores, AL | White | 0.090 | 0.075 | 2048 |
| 467 | 1/25/2014 | 012514-03 | Fort Morgan, AL | White | 0.090 | 0.024 | 2184 |
| 468 | 1/26/2014 | 012614-30 | Gulfport, MS | White | 0.150 | 0.054 | 1925 |
| 469 | 1/26/2014 | 012614-30 | Gulfport, MS | White | 0.150 | 0.054 | 1925 |
| 470 | 1/26/2014 | 012614-37 | Waveland, MS | White | 0.121 | 0.000 | 2144 |
| 471 | 1/30/2014 | 013014-01a | Gulf Shores, AL | Aeppli | 0.191 | 0.080 | 1805 |
| 472 | 1/30/2014 | 013014-01b | Gulf Shores, AL | Aeppli | 0.201 | 0.060 | 1812 |
| 473 | 1/30/2014 | 013014-01c | Gulf Shores, AL | Aeppli | 0.192 | 0.086 | 1795 |
| 474 | 1/30/2014 | 013014-3a | Gulf Shores, AL | Aeppli | 0.164 | 0.078 | 1871 |
| 475 | 1/30/2014 | 013014-3b | Gulf Shores, AL | Aeppli | 0.190 | 0.057 | 1842 |
| 476 | 1/30/2014 | 013014-3c | Gulf Shores, AL | Aeppli | 0.161 | 0.036 | 1951 |
| 477 | 1/30/2014 | 013014-6a | Fort Morgan, AL | Aeppli | 0.166 | 0.052 | 1908 |
| 478 | 1/30/2014 | 013014-6b | Fort Morgan, AL | Aeppli | 0.166 | 0.034 | 1939 |
| 479 | 1/30/2014 | 013014-6c | Fort Morgan, AL | Aeppli | 0.186 | 0.054 | 1857 |
| 480 | 4/25/2014 | 042514-38 | Dauphin Island, AL | White | 0.139 | 0.001 | 2084 |
| 481 | 4/25/2014 | 042514-43 | Dauphin Island, AL | White | 0.107 | 0.000 | 2193 |
| 482 | 4/25/2014 | 042514-44 | Dauphin Island, AL | White | 0.252 | 0.000 | 1779 |
| 483 | 4/25/2014 | 042514-17 | Fort Pickens, FL | White | 0.130 | 0.000 | 2114 |
| 484 | 4/25/2014 | 042514-18 | Fort Pickens, FL | White | 0.154 | 0.000 | 2040 |
| 485 | 4/25/2014 | 042514-32 | Gulf Shores, AL | White | 0.121 | 0.121 | 1857 |
| 486 | 4/25/2014 | 042514-33 | Gulf Shores, AL | White | 0.136 | 0.046 | 1982 |
| 487 | 4/25/2014 | 042514-28 | Perdido Key, FL | White | 0.133 | 0.000 | 2105 |

| | | | | | | | |
|---|---|---|---|---|---|---|---|
| 488 | 4/25/2014 | 042514-29 | Perdido Key, FL | White | 0.167 | 0.000 | 2001 |
| 489 | 4/26/2014 | 042614-32 | Gulfport, MS | White | 0.230 | 0.005 | 1821 |
| 490 | 6/10/2014 | 061014-06 | Fort Morgan, AL | White | 0.127 | 0.070 | 1953 |
| 491 | 6/10/2014 | 061014-08 | Fort Morgan, AL | White | 0.146 | 0.004 | 2056 |
| 492 | 6/10/2014 | 061014-34 | Gulf Shores, AL | White | 0.078 | 0.137 | 1939 |
| 493 | 6/10/2014 | 061014-36 | Gulf Shores, AL | White | 0.085 | 0.003 | 2266 |
| 494 | 6/10/2014 | 061014-36b | Gulf Shores, AL | White | 0.114 | 0.002 | 2162 |
| 495 | 6/10/2014 | 061014-30 | Perdido Key, FL | White | 0.126 | 0.005 | 2115 |
| 496 | 6/11/2014 | 061114-35 | Gulfport, MS | White | 0.196 | 0.021 | 1873 |
| 497 | 6/11/2014 | 061114-60 | West Ship Island, MS | White | 0.075 | 0.006 | 2295 |
| 498 | 6/11/2014 | 061114-61 | West Ship Island, MS | White | 0.095 | 0.001 | 2233 |
| 499 | 6/11/2014 | 061114-62 | West Ship Island, MS | White | 0.075 | 0.004 | 2300 |
| 500 | 6/11/2014 | 061114-63 | West Ship Island, MS | White | 0.107 | 0.058 | 2040 |
| 501 | 6/11/2014 | 061114-39 | Waveland, MS | White | 0.092 | 0.016 | 2201 |
| 502 | 6/11/2014 | 061114-41 | Waveland, MS | White | 0.396 | 0.016 | 1476 |
| 503 | 6/12/2014 | 061214-32 | Elmer's Island, LA | White | 0.047 | 0.004 | 2416 |
| 504 | 6/12/2014 | 061214-34 | Elmer's Island, LA | White | 0.232 | 0.011 | 1804 |
| 505 | 6/12/2014 | 061214-50 | Grand Isle, LA | White | 0.108 | 0.079 | 1985 |
| 506 | 6/12/2014 | 061214-51 | Grand Isle, LA | White | 0.107 | 0.002 | 2190 |
| 507 | 6/23/2014 | 062314-01 | Perdido Key, FL | Reddy | 0.137 | ND | 2059 |
| 508 | 6/23/2014 | 062314-02 | Perdido Key, FL | Reddy | 0.145 | ND | 2032 |
| 509 | 6/23/2014 | 062314-04 | Perdido Key, FL | Reddy | 0.139 | ND | 2053 |
| 510 | 6/23/2014 | 062314-07 | Perdido Key, FL | Reddy | 0.179 | ND | 1934 |
| 511 | 6/23/2014 | 062314-08 | Fort Morgan, AL | Reddy | 0.203 | ND | 1872 |
| 512 | 6/23/2014 | 062314-09 | Fort Morgan, AL | Reddy | 0.098 | ND | 2188 |
| 513 | 6/23/2014 | 062314-10 | Fort Morgan, AL | Reddy | 0.117 | ND | 2122 |
| 514 | 6/23/2014 | 062314-11 | Fort Morgan, AL | Reddy | 0.172 | ND | 1955 |
| 515 | 6/23/2014 | 062314-12 | Fort Morgan, AL | Reddy | 0.191 | ND | 1902 |
| 516 | 6/23/2014 | 062314-13 | Fort Morgan, AL | Reddy | 0.151 | ND | 2015 |
| 517 | 6/23/2014 | 062314-14 | Fort Morgan, AL | Reddy | 0.156 | ND | 1999 |
| 518 | 6/23/2014 | 062314-15 | Fort Morgan, AL | Reddy | 0.131 | ND | 2076 |
| 519 | 6/23/2014 | 062314-16 | Fort Morgan, AL | Reddy | 0.111 | ND | 2141 |
| 520 | 6/23/2014 | 062314-17 | Fort Morgan, AL | Reddy | 0.133 | ND | 2070 |
| 521 | 6/23/2014 | 062314-18 | Fort Morgan, AL | Reddy | 0.201 | ND | 1878 |
| 522 | 6/23/2014 | 062314-19 | Fort Morgan, AL | Reddy | 0.157 | ND | 1998 |
| 523 | 6/23/2014 | 062314-20 | Fort Morgan, AL | Reddy | 0.192 | ND | 1899 |
| 524 | 6/23/2014 | 062314-21 | Fort Morgan, AL | Reddy | 0.198 | ND | 1884 |
| 525 | 6/23/2014 | 062314-23 | Fort Morgan, AL | Reddy | 0.176 | ND | 1943 |
| 526 | 6/23/2014 | 062314-24 | Fort Morgan, AL | Reddy | 0.187 | ND | 1913 |
| 527 | 6/23/2014 | 062314-25 | Fort Morgan, AL | Reddy | 0.149 | ND | 2021 |
| 528 | 6/23/2014 | 062314-26 | Fort Morgan, AL | Reddy | 0.130 | ND | 2080 |

| | | | | | | | |
|---|---|---|---|---|---|---|---|
| 529 | 6/23/2014 | 062314-28 | Fort Morgan, AL | Reddy | 0.161 | ND | 1985 |
| 530 | 6/23/2014 | 062314-29 | Fort Morgan, AL | Reddy | 0.087 | ND | 2226 |
| 531 | 6/23/2014 | 062314-33 | Dauphin Island, AL | Reddy | 0.055 | ND | 2350 |
| 532 | 6/24/2014 | 062414-02 | West Ship Island, MS | Reddy | 0.145 | ND | 2034 |
| 533 | 6/24/2014 | 062414-03 | West Ship Island, MS | Reddy | 0.132 | ND | 2073 |
| 534 | 6/24/2014 | 062414-04 | West Ship Island, MS | Reddy | 0.118 | ND | 2118 |
| 535 | 6/24/2014 | 062414-05 | West Ship Island, MS | Reddy | 0.109 | ND | 2150 |
| 536 | 6/24/2014 | 062414-06 | West Ship Island, MS | Reddy | 0.148 | ND | 2023 |
| 537 | 6/24/2014 | 062414-09 | West Ship Island, MS | Reddy | 0.156 | ND | 2001 |
| 538 | 6/24/2014 | 062414-12 | West Ship Island, MS | Reddy | 0.099 | ND | 2183 |
| 539 | 6/24/2014 | 062414-13 | West Ship Island, MS | Reddy | 0.106 | ND | 2159 |
| 540 | 6/24/2014 | 062414-14 | West Ship Island, MS | Reddy | 0.081 | ND | 2248 |
| 541 | 6/24/2014 | 062414-15 | West Ship Island, MS | Reddy | 0.120 | ND | 2113 |
| 542 | 6/24/2014 | 062414-18 | West Ship Island, MS | Reddy | 0.095 | ND | 2197 |
| 543 | 6/24/2014 | 062414-20 | West Ship Island, MS | Reddy | 0.103 | ND | 2170 |
| 544 | 6/24/2014 | 062414-21 | West Ship Island, MS | Reddy | 0.070 | ND | 2288 |
| 545 | 6/24/2014 | 062414-22 | West Ship Island, MS | Reddy | 0.090 | ND | 2214 |
| 546 | 6/24/2014 | 062414-23 | West Ship Island, MS | Reddy | 0.121 | ND | 2108 |
| 547 | 6/24/2014 | 062414-24 | West Ship Island, MS | Reddy | 0.092 | ND | 2206 |
| 548 | 6/24/2014 | 062414-25 | West Ship Island, MS | Reddy | 0.104 | ND | 2167 |
| 549 | 6/24/2014 | 062414-26 | West Ship Island, MS | Reddy | 0.109 | ND | 2150 |
| 550 | 6/24/2014 | 062414-29 | West Ship Island, MS | Reddy | 0.149 | ND | 2022 |
| 551 | 6/24/2014 | 062414-30 | West Ship Island, MS | Reddy | 0.060 | ND | 2330 |
| 552 | 6/24/2014 | 062414-31 | West Ship Island, MS | Reddy | 0.169 | ND | 1962 |
| 553 | 6/24/2014 | 062414-32 | West Ship Island, MS | Reddy | 0.075 | ND | 2269 |
| 554 | 6/24/2014 | 062414-33 | West Ship Island, MS | Reddy | 0.047 | ND | 2381 |
| 555 | 6/24/2014 | 062414-34 | West Ship Island, MS | Reddy | 0.069 | ND | 2294 |
| 556 | 6/24/2014 | 062414-36 | West Ship Island, MS | Reddy | 0.180 | ND | 1932 |
| 557 | 6/24/2014 | 062414-37 | West Ship Island, MS | Reddy | 0.065 | ND | 2310 |
| 558 | 6/24/2014 | 062414-38 | West Ship Island, MS | Reddy | 0.121 | ND | 2108 |
| 559 | 6/24/2014 | 062414-39 | West Ship Island, MS | Reddy | 0.081 | ND | 2247 |
| 560 | 6/24/2014 | 062414-40 | West Ship Island, MS | Reddy | 0.085 | ND | 2232 |
| 561 | 6/24/2014 | 062414-41 | West Ship Island, MS | Reddy | 0.104 | ND | 2166 |
| 562 | 6/24/2014 | 062414-42 | West Ship Island, MS | Reddy | 0.196 | ND | 1890 |
| 563 | 6/24/2014 | 062414-43 | West Ship Island, MS | Reddy | 0.159 | ND | 1992 |
| 564 | 6/24/2014 | 062414-44 | West Ship Island, MS | Reddy | 0.109 | ND | 2148 |
| 565 | 6/24/2014 | 062414-45 | West Ship Island, MS | Reddy | 0.137 | ND | 2058 |

[a] Reddy - samples collected and processed by Christopher Reddy Lab in Woods Hole, MA.
[b] Aeppli - samples collected and processed by Christoph Aeppli Lab in Boothbay, ME.
[c] White - samples collected and processed by Helen White Lab in Havertown, PA.
[d] ND = No Data
