# Supplementary material for: Hurricane Isaac brings more than oil ashore: Characteristics of beach deposits following the Deepwater Horizon spill
Source: PLoS One. 2019 Mar 18;14(3):e0213464. doi: 10.1371/journal.pone.0213464 (PMC6422254; doi:10.1371/journal.pone.0213464)
Supplement: S2 Table — (PDF) [file pone.0213464.s010.pdf]

S2 Table. Grain size, carbon content and stable carbon isotopic composition ($\delta^{13}C$) of mat samples.

Sample	bulk	> 1 mm	> 500 µm to 1 mm	> 250 to 500 µm	> 150 to 250 µm	< 150 µm
Grain size distribution (%)						
090112-04	--	0.9	8.2	57.4	28.6	5.6
090112-05	--	0.5	8.2	68.8	19.6	3.7
090112-06	--	2.3	6.8	57.3	29.1	5.6
090112-07	--	5.6	8.1	68.6	15.9	2.3
090112-08	--	1.6	7.8	57.2	28.1	6.0
Organic carbon content (wt %)						
090112-04	4.1	29	20	3.5	4.4	19
090112-05	9.3	24	13	2.8	1.5	17
090112-06	7.3	30	18	7.2	4.4	18
090112-07	16	34	26	2.7	4.0	22
090112-08	5.1	36	9.7	2.9	2.6	17
$\delta^{13}C$ values (‰)						
090112-04	-16.2	-17.6	-16.0	-16.7	-16.9	-16.6
090112-05	-15.0	-15.8	-14.9	-16.0	-16.4	-15.6
090112-06	-17.9	-16.9	-15.8	-18.9	-18.2	-18.1
090112-07	-14.4	-13.4	-14.1	-15.6	-15.5	-15.0
090112-08	-19.3	-18.3	-17.8	-19.5	-19.2	-18.2
